# The YfiBNR Signal Transduction Mechanism Reveals Novel Targets for the Evolution of Persistent *Pseudomonas aeruginosa* in Cystic Fibrosis Airways

**DOI:** 10.1371/journal.ppat.1002760

**Published:** 2012-06-14

**Authors:** Jacob G. Malone, Tina Jaeger, Pablo Manfredi, Andreas Dötsch, Andrea Blanka, Raphael Bos, Guy R. Cornelis, Susanne Häussler, Urs Jenal

**Affiliations:** 1 Biozentrum of the University of Basel, Basel, Switzerland; 2 University of East Anglia/John Innes Centre, Norwich Research Park, Norwich, United Kingdom; 3 Helmholtz Center for Infection Research, Braunschweig, Germany; 4 Twincore, Centre of Clinical and Experimental Infection Research, a joint venture of the Hannover Medical School and the Helmholtz Centre for Infection Research, Hannover, Germany; Yale University, United States of America

## Abstract

The genetic adaptation of pathogens in host tissue plays a key role in the establishment of chronic infections. While whole genome sequencing has opened up the analysis of genetic changes occurring during long-term infections, the identification and characterization of adaptive traits is often obscured by a lack of knowledge of the underlying molecular processes. Our research addresses the role of *Pseudomonas aeruginosa* small colony variant (SCV) morphotypes in long-term infections. In the lungs of cystic fibrosis patients, the appearance of SCVs correlates with a prolonged persistence of infection and poor lung function. Formation of *P. aeruginosa* SCVs is linked to increased levels of the second messenger c-di-GMP. Our previous work identified the YfiBNR system as a key regulator of the SCV phenotype. The effector of this tripartite signaling module is the membrane bound diguanylate cyclase YfiN. Through a combination of genetic and biochemical analyses we first outline the mechanistic principles of YfiN regulation in detail. In particular, we identify a number of activating mutations in all three components of the Yfi regulatory system. YfiBNR is shown to function via tightly controlled competition between allosteric binding sites on the three Yfi proteins; a novel regulatory mechanism that is apparently widespread among periplasmic signaling systems in bacteria. We then show that during long-term lung infections of CF patients, activating mutations invade the population, driving SCV formation *in vivo*. The identification of mutational “scars” in the *yfi* genes of clinical isolates suggests that Yfi activity is both under positive and negative selection *in vivo* and that continuous adaptation of the c-di-GMP network contributes to the *in vivo* fitness of *P. aeruginosa* during chronic lung infections. These experiments uncover an important new principle of *in vivo* persistence, and identify the c-di-GMP network as a valid target for novel anti-infectives directed against chronic infections.

## Author Summary

Here we investigate the molecular function of the important cyclic-di-GMP signaling system YfiBNR in the opportunistic pathogen *Pseudomonas aeruginosa* and demonstrate its importance for the evolution of persistent small colony variant (SCV) morphotypes in chronic cystic fibrosis (CF) lung infections. Previously we showed that YfiN is a membrane bound diguanylate cyclase, whose activity is controlled by the soluble periplasmic repressor YfiR and the outer-membrane peptidoglycan binding protein YfiB. In this study we use a combination of genetic and biochemical analyses to investigate the mechanistic principles of YfiN regulation. By examining a series of activating mutations throughout the *yfi* operon, we show that YfiBNR functions via tightly controlled competition between allosteric binding sites on the three Yfi proteins; a novel regulatory mechanism that is apparently widespread among periplasmic signaling systems in bacteria. We then show that during long-term CF lung infections, Yfi activating mutations invade the population, driving SCV formation *in vivo*. The identification of mutational “scars” in the *yfi* genes of clinical isolates further suggests that Yfi activity is both under positive and negative selection *in vivo*, with Yfi-mediated SCVs acting as an environmental pool for the generation of new smooth morphotypes.

## Introduction

*Pseudomonas aeruginosa* is an opportunistic gram-negative pathogen that predominates in late stage cystic fibrosis (CF) lung infections [1]. Once established in the CF lung, *P. aeruginosa* is impossible to entirely eradicate, with repeated relapses of infection and the accompanying aggravation leading to progressive tissue degradation and eventually to death. Over the course of long-term chronic CF lung infections, *P. aeruginosa* undergoes phenotypic and genetic adaptation to the lung environment, resulting in both a progressive transition towards a persistent, low virulence state and a related diversification into a number of distinctive phenotypes [2], [3]. These include mucoid cells, which overproduce alginate and form distinctive slimy colonies [4], and small colony variants (SCVs), slow-growing isolates that show strong attachment to surfaces, auto-aggregation, enhanced exopolysaccharide production and biofilm formation [5], [6]. The appearance of SCVs correlates with a prolonged persistence of infection, poor lung function and increased antibiotic and serum resistance. Fatal systemic infections after lung transplantation and increased serum resistance have been associated with the recovery of SCVs of *Burkholderia* species [7], [8], [9]. *P. aeruginosa* SCVs also emerge in other situations that favor chronic infections including mechanically ventilated patients or patients suffering from chronic obstructive pulmonary disease [8], [10]. These studies suggest that persistent forms of *P. aeruginosa* represent genetic adaptations to the hostile milieu in the patient, with characteristics including resistance to phagocytosis [11], antimicrobial resistance due to slow growth or increased persister cell populations [7], [12], and reduced virulence [13] potentially contributing to selection. Consistent with this, our recent work [11] demonstrated a causal link between *P. aeruginosa* SCVs and persistence of infection in mice, supporting the hypothesis that under certain infection conditions the SCV phenotype confers a fitness advantage, and thus makes an important contribution to the pathogenesis of *P. aeruginosa* lung infections.

In recent years a strong link has emerged between enhanced levels of the second messenger cyclic di-GMP (c-di-GMP) [11], [14], [15], [16] and the SCV phenotype, via overproduction of exopolysaccharides [5], [16] or fimbrial adhesins [14], [17]. C-di-GMP is a ubiquitous and widespread signaling molecule that has been shown to influence a diverse range of cellular processes involved in the transition from a motile, single-cell lifestyle to sessile, surface attached consortia called biofilms [18], [19]. In *P. aeruginosa* c-di-GMP regulates multiple cellular processes, including exopolysaccharide production [20], [21], [22], exposure of fimbrial and proteinaceous adhesins [23], [24], rhamnolipid biosynthesis [25], siderophore production [11], and virulence and cytotoxicity systems [26], [27], [28], as well as assembly and function of pili [29], [30], [31] and flagella [32]. Since many of these cellular processes are subject to phenotypic adaptation during chronic *P. aeruginosa* lung infections, enzymes involved in c-di-GMP metabolism have emerged as possible targets of the underlying genotypic variation [2], [11], [14].

**Figure 1:**
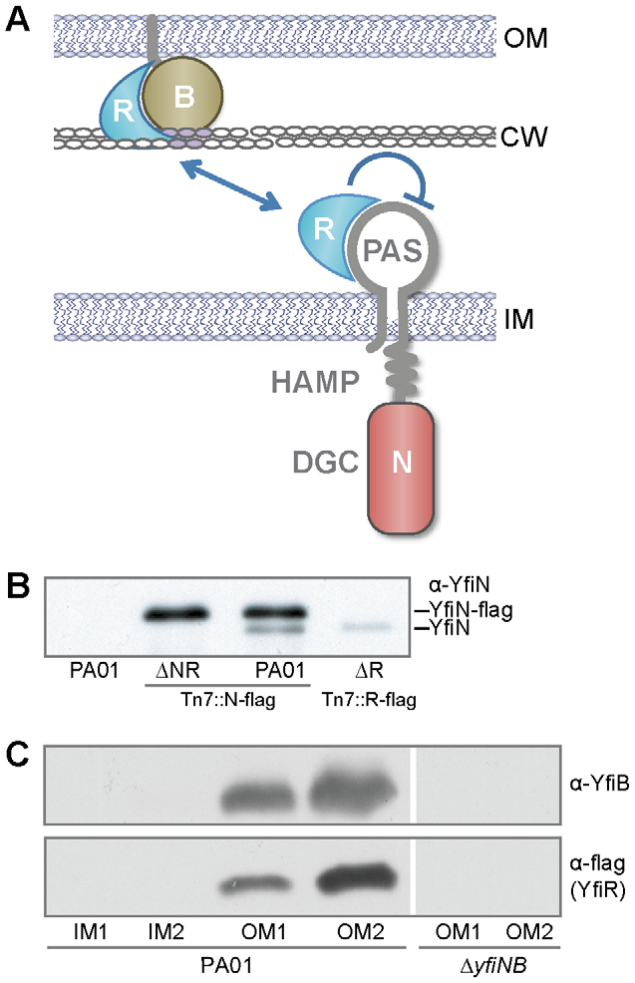
Interactions between the YfiBNR proteins. A) A model of *yfiBNR* interaction. YfiN is a membrane-localized DGC controlled by YfiR. YfiB, the outer-membrane bound Pal-like protein, activates YfiN by sequestering YfiR. B) Co-immunoprecipitation of YfiN with flag-tagged YfiN and YfiR. Immunoblot of boiled M2 resin samples with anti-YfiN antiserum shows YfiN (lower band) co-precipitating with YfiN-flag (upper band) or YfiR-flag. C) Membrane localization of YfiB and YfiR. Immunoblots of fractionated membrane samples with anti-YfiB (upper panel) and M2 antisera (lower 2 panels). The left two panels show membrane fractions for PA01 *yfiR-M2*, the right panel for Δ*yfiBN yfiR-M2*. IM1/2: inner membrane fractions, OM1/2: outer membrane fractions.

YfiBNR [11], [33] (also called AwsXRO [34], [35], [36], TpbB [37]) is a tripartite signaling system that modulates intracellular c-di-GMP levels in response to signals received in the periplasm [11]. The effector of the Yfi system, YfiN, is a membrane integral diguanylate cyclase consisting of a periplasmic PAS domain and cytoplasmic HAMP and catalytic GGDEF domains (Figure 1A). YfiN activity is repressed by the soluble periplasmic protein YfiR and stimulated by the outer membrane lipoprotein YfiB [11]. C-di-GMP produced by YfiN stimulates the production of the Pel and Psl exopolysaccharides, thereby promoting surface attachment in wild-type *P. aeruginosa* and generating an SCV phenotype when YfiN is activated or YfiR repression is released [11]. Homologs of the YfiBNR system are widespread, and have been shown to function similarly in *Escherichia coli*, *Klebsiella pneumonia* and *Pseudomonas fluorescens* SBW25, where they affect biofilm formation through cellulose production [33], [34] or Type 3 fimbriae expression [38].

While the epistasis and mode of output of YfiBNR have been established [11], the mechanistic principles of YfiBNR control remain to be determined. Central unanswered questions include the nature of activating signals and the mechanism of YfiN activation. The periplasmic protein YfiR plays a key role in the signal transduction process as it bridges between the YfiN diguanylate cyclase in the inner membrane and the presumable YfiB sensor in the outer membrane. However, no structural or mechanistic information is available for any members of the YfiR family. In particular, it is unclear how YfiR interacts with the periplasmic PAS domain of YfiN. PAS are versatile domains that activate downstream signaling processes through a range of different mechanisms [39], [40], [41], [42] including ligand binding [41], light/oxygen-driven modification of bound flavin or heme groups [43], [44], homodimerization [45] and, in eukaryotes, heterodimerization [46], [47]. However, given the apparent ubiquity of PAS domains in bacterial signal transduction processes [48] additional activation mechanisms likely exist. Finally, the role and mode of action of YfiB have remained elusive. Epistasis experiments place YfiB upstream of YfiR, and suggest that YfiB activates YfiN by relieving YfiR-mediated repression [11]. YfiB is a structural homolog of Pal, a peptidoglycan binding protein and component of the Tol-Pal pathway required to maintain cell envelope integrity and function [49], [50], [51], [52], although it is unclear whether or not the two proteins are also functional analogs.

In this study we map several adaptive mutations in *P. aeruginosa* SCV isolates from CF patients to the c-di-GMP regulatory *yfiBNR* locus. Moreover, through the elucidation of the signal transduction mechanisms of the YfiBNR system we present a molecular rationale explaining how this system contributes to the evolution of distinct *P. aeruginosa* phenotypes and how the consecutive selection of gain- and loss-of-function *yfiBNR* mutations might contribute to *P. aeruginosa* adaptation in CF lungs. Firstly, a combination of genetic and biochemical analysis was used to produce a detailed molecular map of YfiBNR function. Through the isolation and characterization of “locked-on” and compensatory mutant alleles of all three components of the system, we provide evidence that YfiR inhibits YfiN allosterically, through a hydrophobic interaction between the C-terminus of YfiR and a conserved region of the periplasmic PAS domain of YfiN. Subsequent *in silico* analysis suggests that this YfiR-YfiN interaction represents a novel and widespread periplasmic signaling module, controlling diverse cytoplasmic outputs in a variety of species. YfiN repression is released through an YfiB-dependent sequestration of YfiR to the outer membrane fraction. The resulting structural rearrangements in YfiN are then propagated from the external PAS domain through the transmembrane helices and HAMP domain to activate the C-terminal catalytic GGDEF-domains. Our results suggest that the YfiB lipoprotein that spans the outer membrane and the peptidoglycan acts as a sensor of the YfiBNR system, and may be involved in transducing envelope stress into a rapid increase of c-di-GMP inside the cell and consequent biofilm formation through activation of the Pel and Psl exopolysaccharide systems.

In parallel, screening strategies with two libraries of clinical *P. aeruginosa* strains isolated from CF patients identified a number of SCVs with causal mutations throughout the *yfiBNR* locus. Activating substitutions were found in YfiN and a loss-of-function mutation was isolated in YfiR. In addition, several SCVs harbored mutations in the predicted *yfiBNR* promoter region. The observation that most of these mutations match the “locked-on” mutations that we isolated by *in vitro* genetics strongly argues that *P. aeruginosa* SCVs arise in patients' lungs through genetic alterations that activate the Yfi signaling system. Furthermore, the subsequent identification of clinical isolates containing both *yfi* activating mutations and loss-of-function mutations in *yfiN* suggests that the environment in the lung alternates over time between states that favor and disfavor the formation and fitness of SCVs. Thus, Yfi-mediated SCVs are under positive and negative selection in the dynamic environment of the CF lung thereby enabling *P. aeruginosa* to switch between slow growing and persistent SCVs and fast growing smooth morphotypes.

## Results

### The periplasmic regulator YfiR shuttles between inner and outer membrane

YfiN has previously been shown to function as a membrane bound diguanylate cyclase (DGC) whose activity is repressed by the soluble periplasmic protein YfiR [11], [35]. However, the mechanism of YfiR repression is currently unknown. The simplest potential repression mechanism is allosteric inhibition *via* direct binding of YfiR to the periplasmic PAS-like domain of YfiN. To test whether YfiR interacts with YfiN, co-immunoprecipitation experiments were carried out using an YfiR variant with a C-terminal flag tag. Wild-type YfiN was successfully pulled down by YfiR-flag (Figure 1B), indicating that these proteins interact and that YfiR might function by allosterically inhibiting YfiN activity. Furthermore, YfiN-flag was also shown to interact with YfiN wild type (Figure 1B), indicating that YfiN dimerizes *in vivo*, in common with other diguanylate cyclases [53].

YfiB is predicted to be an outer membrane lipoprotein on the basis of primary structure analysis and the fact that the protein is found exclusively in the insoluble fraction of lysed PA01 [11]. This was confirmed by membrane fractionation experiments that located YfiB exclusively in the outer membrane fractions (Figure 1C). Although co-immunoprecipitation experiments failed to show direct interaction between YfiB and YfiR (data not shown), YfiR-flag localization to the outer membrane fraction is strictly YfiB-dependent. In the absent of YfiB, YfiR-flag is stable (Figure S1B), but no longer associates with the membrane (Figure 1C, S1A).

### Isolation of constitutive mutants delineates the mode of YfiN diguanylate cyclase activation

**Figure 2:**
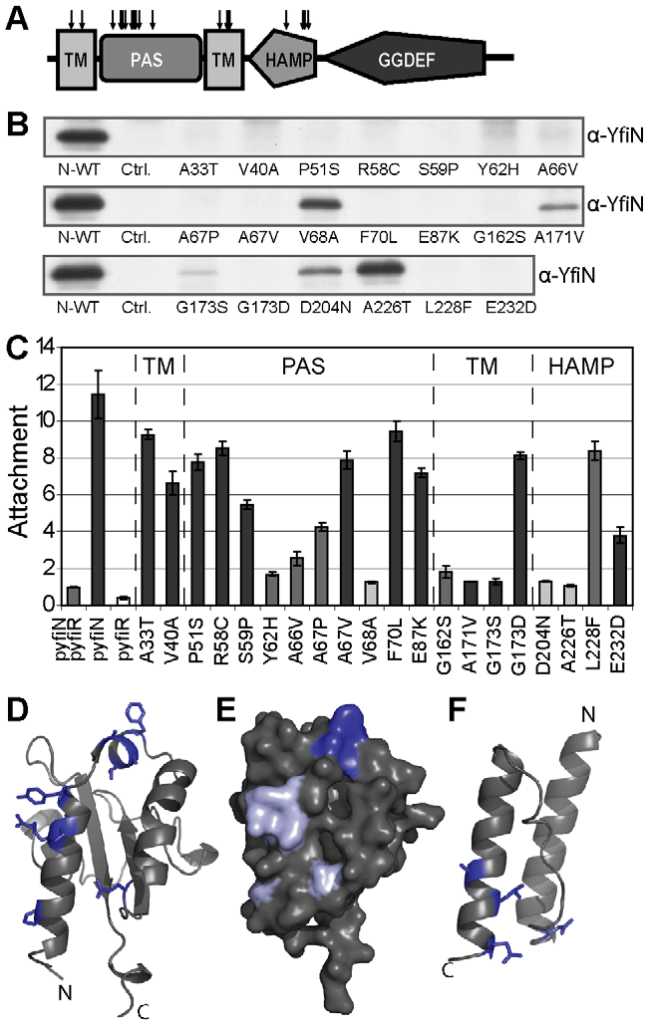
Activating mutations in YfiN. A) Locations of activating mutations in YfiN. Arrows indicate the positions of activating mutations throughout the structure of YfiN. TM: transmembrane helix. The cartoon is drawn to scale. B) Co-immunoprecipitation of active YfiN alleles with flag-tagged YfiR. Immunoblot of boiled M2 resin samples with anti-YfiN antiserum. N-WT shows the *DyfiNR* screening strain with both *yfiN* (WT) and *yfiR-flag* plasmids present. Ctrl. shows *ΔyfiNR* with pGm*-yfiprom-N* only. The point mutation present in YfiN is indicated for the remaining lanes, which show YfiN immunoprecipitated from *ΔyfiNR* strains containing both *yfiR-flag* and the mutated *yfiN* plasmids. C) Attachment of the active YfiN alleles is shown relative to *ΔyfiNR* pGm*-yfiprom-N*, pMR*-yfiR-flag* (p-*yfiN* p-*yfiR*). Controls containing the *yfiN* or *yfiR-flag* plasmid only are also shown. The point mutation present in YfiN is indicated for each bar. Light grey bars indicate mutants whose activity was compensated for by mutations at the C-terminus of YfiR. Mutants with mid grey bars were compensated for by mutations in the signal sequence, or by uncharacterized mutations, while those with dark grey bars were not compensated for during YfiR mutagenesis. The domain locations of mutants are indicated with TM, PAS etc. D) Cartoon showing the locations of activating substitutions (blue) on a homology model of the YfiN PAS domain (residues 44–154). The PAS model is based on the CitA periplasmic domain (see Materials and methods). E) Surface representation of the YfiN PAS model. The locations of activating mutants on the proposed homodimer interface are shown in light blue, those at the possible YfiR binding site are shown in dark blue. F) The locations of activating substitutions (blue) on a homology model of the YfiN HAMP domain (residues 183–236). N and C termini are marked in D) and F). The HAMP model is based on the Aer2 HAMP structure (see Materials and methods).

If YfiR represses YfiN activity through direct binding to its periplasmic PAS domain, it should be possible to isolate constitutively active YfiN variants that fail to bind YfiR. The positions of these activating residue substitutions would consequently provide insights into the mechanism of YfiN function and the binding interface of YfiN and YfiR. Previously, similar experiments have been successfully used to probe the structure-function relationship of the *P. fluorescens* DGC WspR [54], [55]. To identify YfiR-insensitive YfiN alleles, a screening system was designed in which *yfiN* and *yfiR-flag* are expressed from two separate plasmids in a Δ*yfiNR* background (see Materials and Methods). A pool of *yfiN* variants was produced by XL-1 red mutagenesis of the *yfiN* plasmid and screened for mutants that induced an SCV phenotype in the Δ*yfiNR* tester strain containing a plasmid-borne copy of *yfiR*. Sequencing identified the locations of twenty independent, activating *yfiN* mutations. Two residues were identified in the first transmembrane helix, ten were located towards the N-terminal end of the periplasmic PAS domain, four were found in the second transmembrane helix, and four towards the C-terminal end of the HAMP domain (Figure 2A). No mutations were found in the GGDEF domain. Since most of these mutations were isolated several times independently, we assume that the screen was approaching saturation (Table 1).

**Table 1:** YfiR-insensitive YfiN alleles.

Location in YfiN	Residue change	Notes
First TM helix	A33T	
	V40A	
PAS, dimerization interface	P51S	
	R58C	
	S59P	Equivalent mutation activates AwsX [35].
	Y62H	
	A66V	
	A67P	
	A67V	
	V68A	
	F70L	
	E87K	Mutation present in Clin110.
Second TM helix	G162S	
	A171V	
	G173S	
	G173D	Mutation present in SCV20265.
HAMP	D204N	Mutation at the equivalent position activates Tsr [59].
	A226T	
	L228F	
	E232D	Mutation at the equivalent position activates NarX [58].

PAS refers to the periplasmic PAS domain; HAMP refers to the HAMP domain.

Co-immunoprecipitation experiments showed that most of the activated YfiN alleles no longer bind to YfiR-flag (Figure 2B). In five cases (V68A, A171V, G173S, D204N, and A226T), residual YfiN-YfiR binding was still observed. In accordance with this, these five mutants produced the mildest phenotypes, with relatively low levels of surface attachment (Figure 2C) and partial SCV colony morphologies (Figure S2A). Expression of these five *yfiN* alleles in a *ΔyfiNR* strain produced a distinctive SCV phenotype (data not shown), indicating that the weaker SCV morphology seen with these alleles is likely due to partial inhibition by YfiR, rather than loss of YfiN function. The observation that activating mutations in YfiN abolished YfiR binding independently of their position within the protein (Figure 2B), suggested that the protein switches between discrete active and inactive states, and that the YfiR binding site is obscured in the active conformation.

A clearer suggestion of how YfiN functions was obtained when the positions of the YfiR-insensitive mutations were marked on homology models of the PAS and HAMP domains of YfiN. Activating mutations in the PAS domain appear to cluster into two regions of the 3-D structure modelled on the periplasmic domain of the sensor kinase CitA [41]. Assuming a similar dimerization mechanism for YfiN, the first group of substitutions would cluster at the interface between the two protein monomers (Figure 2D, 2E pale blue), where they might help to stabilize the active YfiN conformation. The remaining substitutions cluster on the domain surface most distal from the inner membrane. Three of these residues (Ala66, Ala67 and Val68) coordinate the position of the fourth (Phe70), which protrudes into the periplasmic space. These residues are predicted to form an exposed hydrophobic patch on the surface of the PAS domain, which we propose as a possible YfiR binding site (Figure 2E; dark blue). The four mutations in the HAMP domain lie close to one another and distal to the inner membrane (Figure 2F). Three mutations (positions 226, 228 and 232) are adjacent to residues required for helical bundle formation [56]. Specific substitutions at these positions may act to stabilise an active HAMP conformation relative to the inactive structure [56], [57]. In support of this, a substitution at the equivalent position to Glu232 in NarX renders the protein constitutively active [58]. The D204N mutation occupies an equivalent position in the helical linker region to the structurally important Leu237 residue of the *E. coli* serine-specific chemoreceptor Tsr. Mutations in this position have been shown to stabilise the activated form of Tsr [59], [60], suggesting a similar mechanism for the YfiN mutation.

Taken together, these results demonstrate that YfiN activation is crucially dependent on a number of key residues involved in intra-molecule signal transduction and on interfering with YfiR binding. We propose that the release of YfiR results in a conformational shift of the entire YfiN protein towards an active state, in which binding of the repressor is disfavored.

### Compensatory mutations cluster in defined regions of the YfiR protein

**Figure 3:**
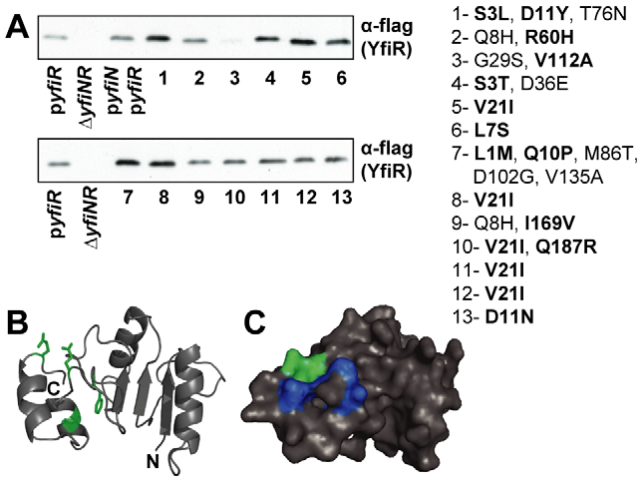
Compensatory YfiR alleles. A) Immunoblots with M2 antiserum, showing levels of compensatory YfiR-flag variants in whole cell lysates. p*yfiR*: *ΔyfiNR* pMR*-yfiR-flag*, Δ*yfiNR*: strain without vector, p*yfiN*/p*yfiR*: *ΔyfiNR* pGm*-yfiprom-N*, pMR*-yfiR-flag*, lanes 1–13: Δ*yfiNR* pGm*-yfiprom-N* with compensatory pMR*-yfiR-flag* plasmids, proposed activating mutations are highlighted in bold. Mutants in lane 1, 4–8, 10–13 harbor mutations in the signal peptide that enhance expression, see also Table 2. B) Cartoon showing the locations of activating substitutions (green) on a homology model of YfiR (comprising residues 68–190). N and C termini are marked. The YfiR model is based on multiple structures (see Materials and methods). C) Surface representation of the YfiR model. The locations of activating mutants are shown in green, hydrophobic residues forming the possible YfiN binding surface are shown in dark blue.

To probe the YfiN-YfiR interaction in more detail, we undertook a screen for compensatory *yfiR* alleles, i.e. alleles that would restore wild-type (WT) colony morphology in the presence of some of the activated YfiN variants introduced above. Following PCR mutagenesis of *yfiR*, twenty-one alleles were isolated across eight *yfiN* mutant backgrounds (Table 2). Several *yfiR* alleles were independently isolated in several constitutive *yfiN* backgrounds, resulting in a total of fourteen unique, compensatory *yfiR* alleles, most of which cluster in the secretion signal sequence or in the C-terminal region of the protein (Table 2, Figure 3B). As the signal peptide is cleaved following export of YfiR into the periplasm, mutations in this region are not predicted to affect the final protein structure. Rather, these mutations might boost YfiR levels in the periplasm through increased translation or export of the protein. This was confirmed by immunoblot analysis demonstrating that signal peptide mutants indeed produced higher overall levels of YfiR than wild-type (Figure 3).

**Table 2:** Compensatory YfiR alleles.

YfiN allele	No.	YfiR residue change(*)	Location in YfiR
Y62H	12	**V21I**	Signal peptide
	13	**D11N**	Signal peptide
A66V	1	**S3L**, **D11Y**, T76N	Signal peptide
A67P	10	**V21I**, **Q187R**	Signal peptide/proposed YfiN binding site
V68A	-	R93S, **I169V**	proposed YfiN binding site
	-	**E163G**	proposed YfiN binding site
	-	**Q187R**	proposed YfiN binding site
	3	G29S, **V112A**	undefined
	4	**S3T**, D36E	Signal peptide
	5	**V21I**	Signal peptide
	6	**L7S**	Signal peptide
G162S	-	**Q125L**	undefined
D204N	-	**K63E**	conserved region of YfiR
	-	**I169V**	proposed YfiN binding site
	11	**V21I**	Signal peptide
A226T	-	**E163G**	proposed YfiN binding site
	7	**L1M**, **Q10P,** M86T, D102G, V135A	Signal peptide/improved start codon
	8	**V21I**	Signal peptide
	-	**F151L**	proposed YfiN binding site
	9	Q8H, **I169V**	proposed YfiN binding site
L228F	2	Q8H, **R60H**	conserved region of YfiR

(*): proposed activating mutations are highlighted in bold.

Compensatory mutations in the C-terminus of YfiR were isolated in three distinct *yfiN* mutant backgrounds, all of which produced mild SCV phenotypes and showed residual binding of YfiR (Table 1, Figure 2). This suggests that mutations in this region of YfiR enhance binding to YfiN, but cannot compensate for a total loss of protein-protein interaction. In a cross-complementation experiment, all four C-terminal YfiR mutants (F151L, E163G, I169V and Q187R) were able to suppress the SCV phenotype of all three weak YfiN mutants (Figure S2B), indicating that the enhanced effectiveness of these mutants was the result of an overall increase in YfiR binding affinity, rather than through complementation of specific YfiN mutations. When plotted onto a homology model of YfiR, the four C-terminal mutations surround a hydrophobic region on the surface of the model (Figure 3B) that presents a possible candidate for the YfiN binding surface. These residues are highly conserved among YfiR homologs, especially the central Phe residue at position 162. Likewise, despite little overall conservation of the YfiN PAS domain, the predicted YfiR binding site on its surface (‘AAVVF’ motif) is highly conserved, but absent in the PAS domain of CitA [41], [61] (see below).

A plausible model for YfiN-YfiR interaction arises from these observations, in which the exposed phenylalanine on the surface of YfiN is hidden from the aqueous environment by hydrophobic interaction with the C-terminus of YfiR. Together, these data demonstrate that activating *yfiN* alleles can be overcome by compensatory mutations in *yfiR*, again lending support for a direct repression of YfiN by the periplasmic protein YfiR.

### Genetic dissection of the YfiB outer membrane sensor

**Figure 4:**
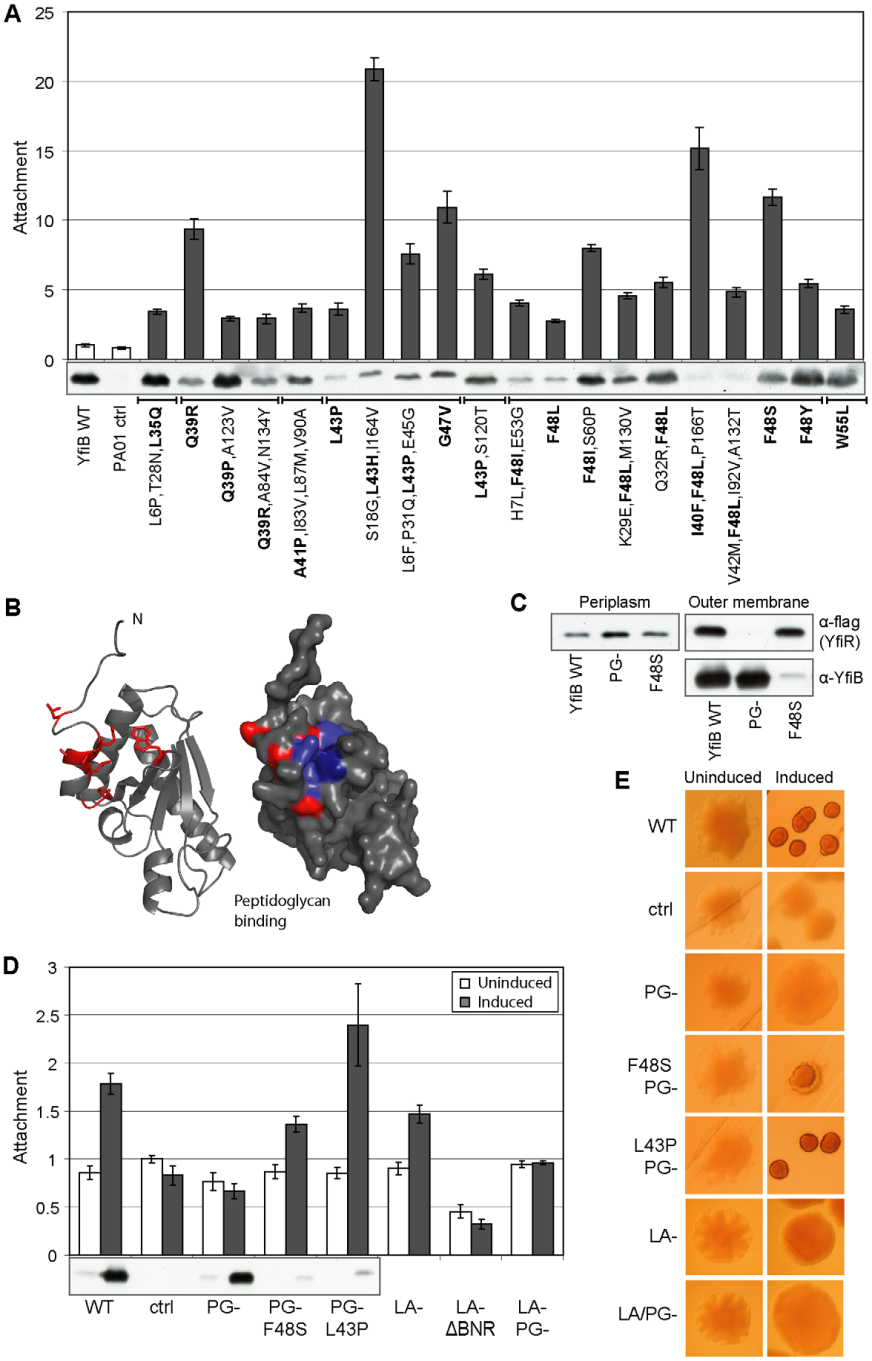
Mutational analysis of YfiB. A) The effect of activating *yfiB* mutants, expressed from pME6032 in Δ*yfiBNR* Tn*7*::*yfiNR*, on attachment is shown relative to PA01 (PA01 ctrl.). ‘YfiB WT’ indicates pME6032*-yfiB*. The point mutants in YfiB are indicated for the remaining lanes. Those mutations thought to contribute to YfiB activation are marked in bold. The immunoblot shows the levels of YfiB protein present in each strain. B) Left: Cartoon showing the locations of activating substitutions (red) on a homology model of YfiB (comprising residues 27–168). The YfiB model is based on the Omp/Pal structure (see Materials and methods for details). The N terminus and peptidoglycan binding site are marked. Right: Surface representation of the YfiB model. The locations of activating mutants are shown in red, hydrophobic residues forming the possible YfiR binding surface are shown in dark blue. C) Co-localization of YfiB and YfiR at the outer membrane. Immunoblots of fractionated soluble and membrane samples with anti-YfiB and M2 antisera as shown. ‘YfiB WT’ indicates Δ*yfiBNR* Tn*7*::*yfiR-flag* containing pME6032*-yfiB*. ‘PG-’ indicates the same background strain containing pME-*yfiB-PG-* (YfiB containing the D102A and G105A substitutions), while ‘F48S’ contains the hyperactive *yfiB F48S* plasmid. D) The effect of different *yfiB* mutants, expressed from pME6032 in Δ*yfiBNR* Tn*7*::*yfiNR*, on attachment is shown relative to pME6032 only (ctrl.). ‘PG-’ mutants ± F48S or L43P mutations as indicated. In ‘LA-’ mutants, the lipid anchor is missing, the signal peptide has been replaced with that from YfiR. ‘Δ*BNR* LA-’ indicates the *yfiB LA-* mutant in the *ΔyfiBNR* strain background. The immunoblot shows the levels of YfiB protein present in each strain. E) Colony morphologies on LB Congo-red agar upon over-expression of *yfiB* mutants.

YfiB is predicted to be an outer-membrane lipoprotein with a PAL-like peptidoglycan (PG) binding domain. Overproduction of YfiB leads to YfiN-dependent SCV formation [11]. How this effect is exerted on YfiN is not clear and no detailed model for YfiB function in *P. aeruginosa* exists. To investigate the function of YfiB, a screen was conducted for activating mutants that induced an SCV phenotype in PA01 without overproduction of the protein. A total of 20 *yfiB* alleles were isolated, each containing one or more amino acid substitutions. All activating *yfiB* alleles caused increased surface attachment and biofilm formation (Figure 4A). Strikingly, while mutations were distributed throughout the sequence of *yfiB*, at least one substitution was found between residues 35 and 55 in all cases. These affected a total of seven positions, five of which were also isolated as single activating substitutions (Figure 4A). Alleles with single mutations in the YfiB N-terminus generally had strong effects that were weakened by the presence of secondary mutations (for example, YfiB L43P produces higher attachment than L43P alleles containing additional mutations). Together this argued that the majority of mutations leading to YfiBNR activation cluster in this region of the YfiB protein. Some of these variants had very strong activating effects despite showing severely reduced stability (Figure 4A). Three further, similar substitutions (I40F, V42M, and E45G) were found in conjunction with other singly isolated activating mutations. While neither the V42M nor the E45G substitutions contributed positively to YfiB activity, the *YfiB*-I40F-F48L allele produced a far stronger phenotype than F48L alone, suggesting that the I40F mutation also contributes to YfiB activation (Figure 4A). When the locations of the activating mutations were plotted onto a 3-D homology model of YfiB, they clustered around the first helix of the PAL domain (Figure 4B). Interestingly, activating residues in YfiB surround a predicted surface-exposed hydrophobic region, similar to that seen in the model of YfiR (Figure 3C). This hydrophobic patch is highly conserved in YfiB homologs, but absent in the YfiB structural homolog OprL [61], [62]. Most notably, W55, predicted to form the core of the hydrophobic binding site, is very highly conserved, only replaced in a small minority of cases with phenylalanine (note that the YfiB W55L mutation forms a weak SCV in PA01).

To determine whether binding to PG is important for YfiB function, mutants were constructed with two critical peptidoglycan-binding residues [63] replaced with alanine. Expression of the resulting gene, *yfiB-D102A-G105A* (PG-) did not induce an SCV colony morphology and had no effect on attachment, despite producing wild type-like levels of protein (Figure 4D, E). To assess the role of the YfiB OM lipid anchor (LA), the YfiB signal peptide was replaced with that from YfiR, which lacks the Cys lipid acceptor residue. The resulting mutant (LA-) slightly increased attachment upon induction in an YfiN-dependent manner (Figure 4D). This residual activity was dependent on peptidoglycan binding, as a PG-/LA- mutant was fully inactive (Figure 4D). These data suggest that peptidoglycan binding and, to a lesser extent, membrane anchoring are required for YfiB activity. When the mutations disrupting PG binding were combined with activating mutations in *yfiB* (F48S or L43P; see above), very low YfiB protein levels were detected, possibly as a result of reduced protein stability (Figure 4D). Importantly, the resulting alleles still led to increased attachment and generated an SCV phenotype (Figure 4D, E). This suggested that the activating mutations are dominant over the loss of peptidoglycan binding, and that they are able to fix YfiB in its active conformation independent of PG binding.

YfiB sequesters YfiR at the outer membrane (Figure 1). To examine the relationship between this activity and YfiB function, membrane fractionation was carried out for strains expressing PG- and hyperactive (F48S) YfiB variants alongside an *yfiR-flag* allele. While wild-type YfiB and the constitutive mutant F48S were able to sequester YfiR to the outer membrane fraction, no localization was seen for the PG binding mutant of YfiB (Figure 4C). Together, these data suggest that YfiB is able to release YfiN repression by sequestering YfiR at the outer membrane and that this activity requires both YfiB peptidoglycan binding and anchoring in the outer membrane. Since we were unable to co-immunoprecipitate YfiB and YfiR, it is unclear if YfiB sequesters YfiR through direct protein-protein contact or if additional component(s) mediate this interaction.

**Figure 5:**
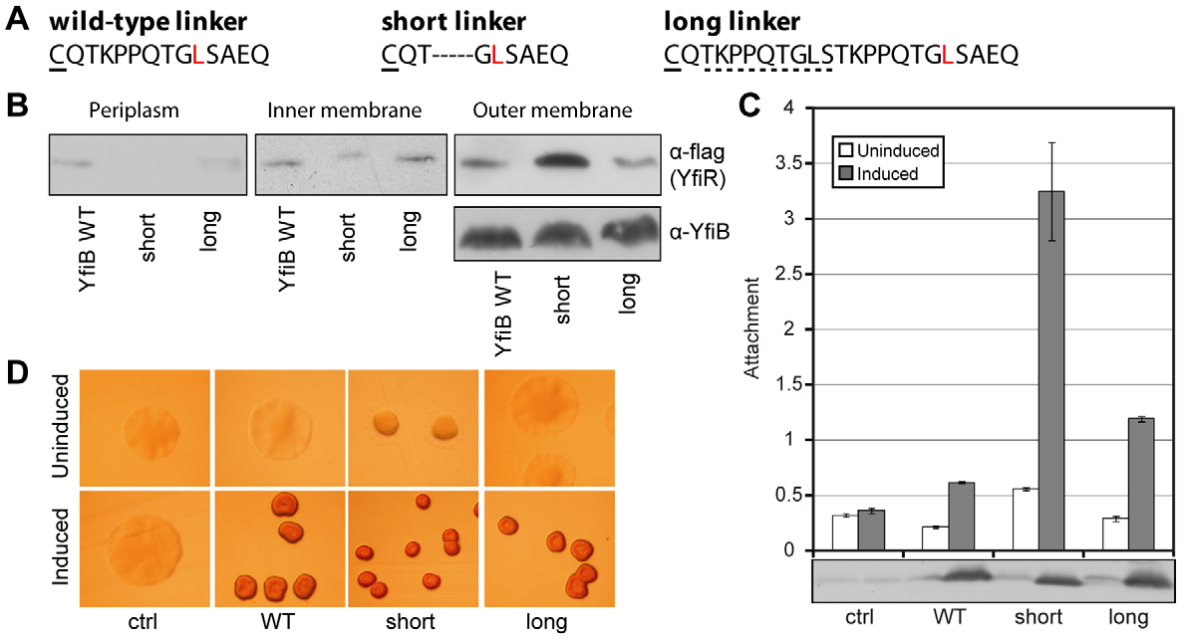
Effect of YfiB linker mutants. A) Sequence of the N-terminal YfiB ‘linker’, between the outer membrane (lipid anchor on the highlighted cysteine) and the Pal-like domain. The first activating mutant position (Figure 4) is shown in red. ‘short-linker’ indicates a deletion of 5 amino acids, leaving the lipobox intact. ‘long-linker’ indicates an insertion/duplication of 9 amino acids (dashed line). B) Co-localization of YfiB and YfiR at the outer membrane. Immunoblots of fractionated soluble and membrane samples with anti-YfiB and M2 antisera as shown. ‘YfiB WT’ indicates Δ*yfiBNR* Tn*7*::*yfiNR* containing pME6032*-yfiB*. ‘short’/‘long’ indicate the strains containing pME6032-*yfiB* linker mutants. C) The effect of different *yfiB* mutants, expressed from pME6032 in Δ*yfiBNR* Tn*7*::*yfiNR*, on attachment is shown relative to pME6032 only (ctrl.). The immunoblot shows the levels of YfiB protein present in each strain. D) Colony morphologies on LB Congo-red agar upon over-expression of *yfiB* mutants.

The finding that both lipid anchor and peptidoglycan binding are required for full activity of YfiB, together with the mapping of gain-of-function mutations close to the YfiB N-terminus, prompted us to examine the role of the N-terminal linker region in YfiB mediated signaling. The need for YfiB anchoring in cell wall and outer membrane suggests that YfiR sequestration might respond to the distance spanned by the protein. To test this, we modulated the length of the 13-amino acid long linker connecting the lipid acceptor cysteine with the PAL domain (Figure 5A). While extending the linker by 9 amino acids produced only a modest attachment effect upon induction (Figure 5C), shortening the linker by 5 amino acids produced a strong constitutive phenotype with respect to YfiR sequestration to the outer membrane (Figure 5B), surface attachment (Figure 5C), and SCV morphology (Figure 5D). This suggests that the linker region plays an important role in YfiB-mediated signaling and that YfiR sequestration might critically depend on the ‘wingspread’ of the YfiB connector between the two outermost layers of the cell.

### YfiBNR as a potential periplasmic stress sensor system

The above experiments, together with the observation that the YfiB homolog Pal is involved in cell envelope homeostasis and function [64] argued that YfiB might induce an up-regulation of c-di-GMP levels in response to cell envelope stress. Consistent with this, the YfiBNR system plays a role in the cellular response to elevated salt concentrations (high osmolarity) and exposure to outer-membrane disturbing detergents like SDS (Figure S3). However, while such a mechanism is consistent with our experiments suggesting that YfiB envelope anchoring is important for its regulatory role, attempts to link YfiB to distinct forms of envelope stress have so far remained unsuccessful.

**Figure 6:**
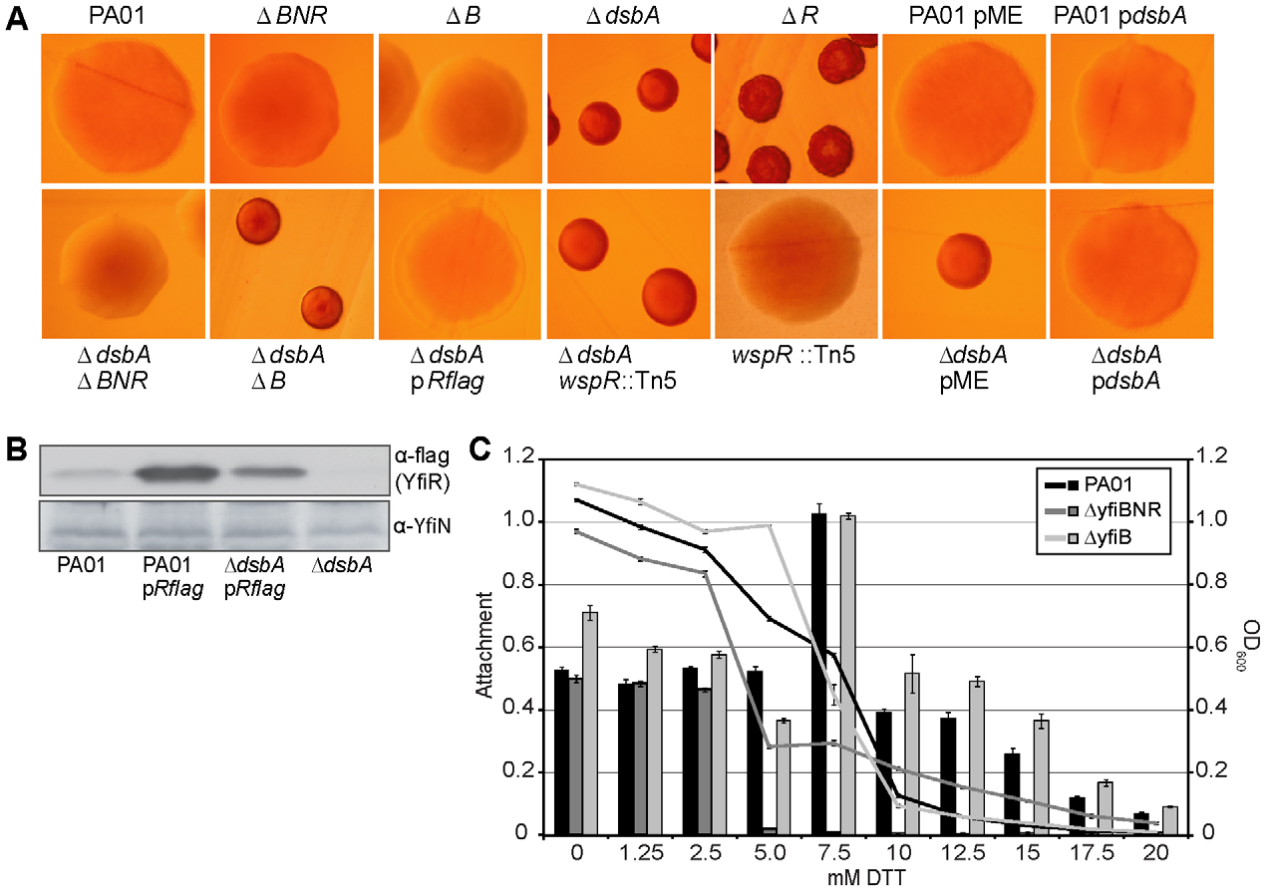
Reducing effects on *yfiBNR* activity. A) Colony morphologies of *yfiBNR/dsbA* single and double mutants on LB Congo-red agar. Indication of the following abbreviations: Δ*BNR* – Δ*yfiBNR*, Δ*B –* Δ*yfiBNR* Tn*7*::*yfiNR*, Δ*R* – Δ*yfiR*, pR-flag – pMR*-yfiR-flag*, p*dsbA* – pME6032ara-*dsbA*, pME – pME6032ara. B) Immunoblots of PA01 and Δ*dsbA* with M2 and anti-YfiN antisera, showing levels of YfiR-flag and YfiN in whole cell lysates. C) The effect of increasing concentrations of DTT on attachment (bars), of the Δ*BNR* and Δ*B* mutants is shown relative to wild-type PA01. Curves represent relative optical density with standard errors.

*In silico* analysis of available bacterial genome sequences revealed that the Yfi signaling family is widespread in gram-negative bacteria and that several of its members lacked the respective YfiB component (see below). This argued for an additional level of signal input within this regulatory network. In a previous screen for mutants leading to an SCV phenotype [11], we identified transposon insertions in *PA5489* that codes for the periplasmic thiol:disulfide interchange protein DsbA. This protein catalyzes cysteine crosslinking, and is responsible for the correct folding of periplasmic proteins [65]. Given that YfiR contains four highly conserved cysteines, we hypothesised that disruption of DsbA may result in YfiR misfolding and consequent release of YfiN repression (YfiN contains no periplasmic cysteines). To test if the *dsbA* SCV phenotype was dependent on the Yfi system, *dsbA* was deleted in PA01 wild type and in different *yfi* mutant strains. Deletion of *dsbA* produced a strong SCV phenotype in PA01 and in the *yfiB* mutant, but not in the Δ*yfiBNR* strain (Figure 6A). Other phenotypes associated with *dsbA* deletion such as a reduction in secreted protease levels were unaffected in the Δ*yfiBNR* background (data not shown). Also, disruption of the *wspR* gene encoding another major *P. aeruginosa* diguanylate cyclase [15], did not abrogate the SCV phenotype of the Δ*dsbA* background (Figure 6A). Consistent with a lower YfiR stability resulting from protein misfolding, YfiR levels, but not YfiN levels, were markedly reduced in the Δ*dsbA* background as compared to wild-type PA01 (Figure 6B). Finally, the Δ*dsbA* SCV phenotype could be abolished by over-expression of *yfiR-flag in trans* (Figure 6A,B). Altogether, this suggested that in the absence of DsbA, misfolding of YfiR leads to specific activation of the YfiN diguanylate cyclase.

The observation that YfiR activity requires DsbA suggested that reducing conditions in the periplasm might block YfiR and lead to YfiN activation. To test this, growth and surface attachment were scored for various PA01 mutants with increasing concentrations of the reducing agent DTT. As shown in Figure 6C, deleting *yfiBNR* markedly increased the sensitivity of PA01 to reducing conditions. While the *yfi* ^+^ strain showed attachment up to 20 mM DTT, the *yfi* mutant showed no attachment above 2.5 mM DTT. Importantly, deletion of *yfiB* alone had no appreciable effect on PA01 growth or attachment in DTT, strongly suggesting that the activation of YfiN under reducing conditions is a consequence of YfiR misfolding rather than signaling through YfiB (Figure 6C). Together, these data suggest that YfiR constitutes an YfiB-independent sensing device that is able to activate the YfiN diguanylate cyclase in response to the redox status of the periplasm.

### The YfiBNR system is under positive and negative selection in CF lungs

“In vitro” mutagenesis of the *yfiBNR* operon has revealed several routes to Yfi-mediated SCV formation. Disruption of *yfiR* induces a strong SCV phenotype [11], and activating mutations can arise in *yfiN* or *yfiB* (Figure 2, 4). To determine whether mutations in the *yfi* genes were responsible for the SCV phenotypes of clinical *P. aeruginosa* isolates from CF patients, we sequenced the *yfiBNR* operon of clinically derived SCVs and analyzed the contribution of candidate SNPs. Firstly, 45 clinical SCV strains were pooled and their genomes sequenced (S. Häussler and A. Dötsch, unpublished data). The *yfiBNR* operon sequence of each pool (representing a consensus sequence drawn from 15 individual SCV genome sequences) was compared with that of PA01, the positions of SNPs were identified (Table S3), and non-synonymous SNPs reproduced and introduced into the SCV screening strains described above (for details see Materials and Methods). From a total of 11 mutations identified, seven showed no noticeable effect. However, several mutations were identified in *yfiN* (G173D, D223N, L227M) or *yfiR* (C26R) that induced an SCV morphology and greatly enhanced attachment in the screening strain (Figure 2C, 7A, D). The amino acid residues altered in YfiN either matched (G173) or were located in the immediate vicinity of positions (D223, L227) identified by *in vitro* mutagenesis (Figure 2). These data demonstrate that SCVs arise in the airways of CF patients through the selection of clones carrying mutations in *yfi* genes that lead to de-repression the Yfi pathway.

**Figure 7:**
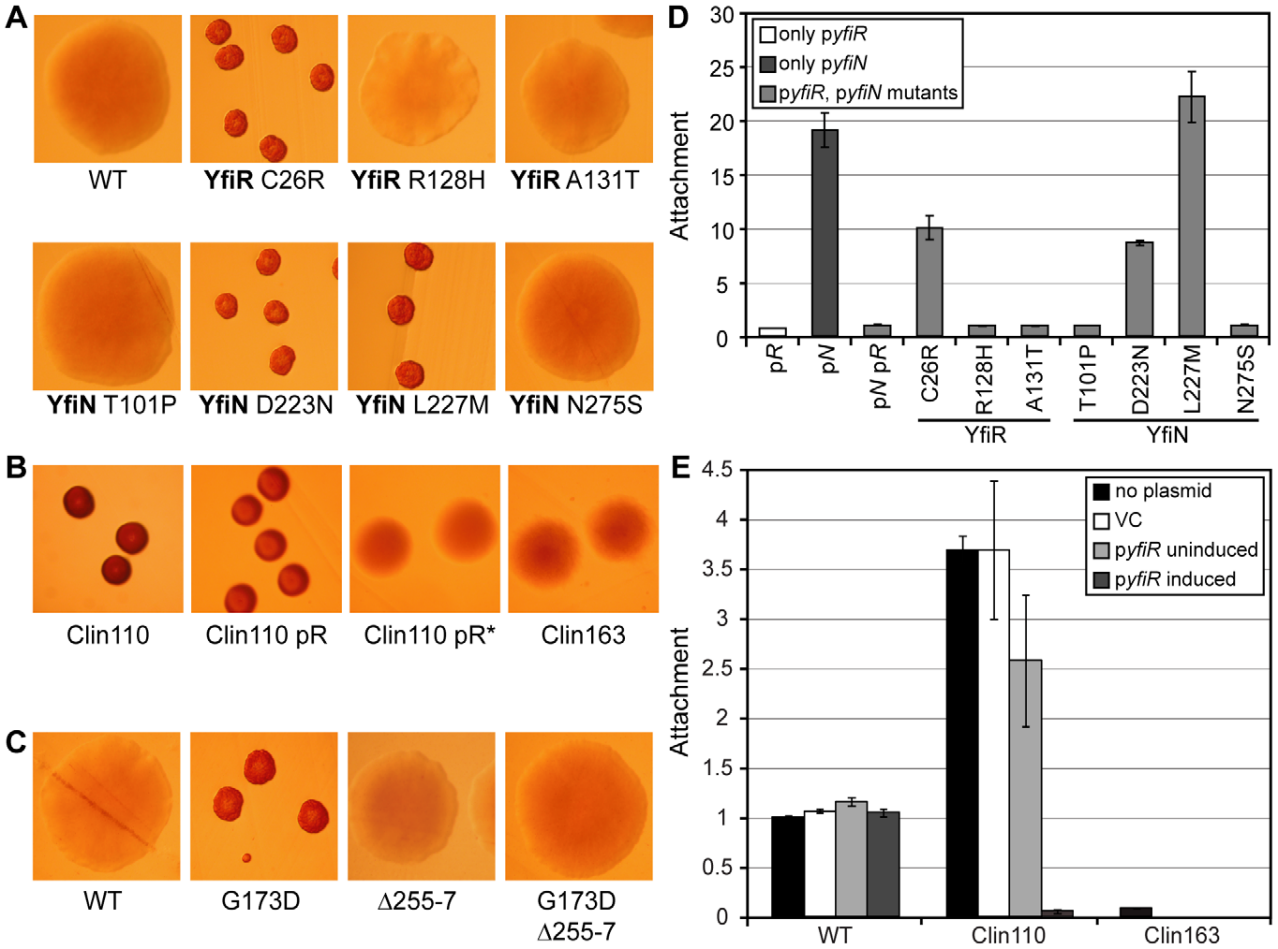
Clinical YfiBNR mutants. A) Colony morphologies of *ΔyfiNR* pGm*-yfiprom-N*, pMR*-yfiR-flag* strains with the point mutants indicated present in either YfiR or YfiN. ‘WT’: both plasmids contain wild-type *yfiN/yfiR* alleles. All colony morphologies are on LB Congo-red agar. B) Colony morphologies of Clin110 and Clin163 strains. pR indicates that the strain contains pME-araC-*yfiR*, the asterisk indicates induction. C) Colony morphologies of *ΔyfiBNR* strains complemented with the *yfiBNR* operon inserted into the *att*-Tn*7* site. Mutations in the complementing copy of *yfiN* are indicated in each case. ‘WT’: wild type *yfiN*. D) Attachment relative to *ΔyfiNR* pGm*-yfiprom-N*, pMR*-yfiR-flag* (pNpR) of clinical *yfiN/yfiR* mutants. ‘pR’ and ‘pN’ contain pMR*-yfiR-flag* or pGm*-yfiprom-N* only. E) Attachment of Clin110 ± pME-araC-*yfiR* and Clin163 relative to PA01.

Next, we analyzed two clinical SCV strains in more detail. First, the SCV morphology and strong surface attachment of strain Clin110 were suppressed by overexpression of *yfiR in trans* (Figure 7B, E). Sequence analysis revealed that Clin110 YfiN contained the activating mutation E87K (Figure 2; Tables 1, S3). Interestingly, a second isolate (Clin163) was recovered 18 months later from the sputum of the same patient and identified as a descendent of Clin110 by comparison of synonymous SNPs throughout the *yfiBNR* operon. Clin163 displayed a smooth colony morphology and low surface attachment (Figure 7B, E), in spite of the fact that it contained the *yfiN* E87K mutation. Clin163 harbored an additional mutation (G329C) in the GGDEF active site motif of the diguanylate cyclase domain. As such active site mutations are known to destroy enzyme activity [54], this strongly suggested that the recovery of smooth colony morphology in Clin163 was due to YfiN inactivation (Figure 7B). In support of this, the c-di-GMP level in Clin110 (1517±280 pmol/mg protein) was measured, and shown to be ∼30 times higher than in PA01 (51.5±20.7). The level in Clin163 in contrast, was much lower (114±41.6). Finally, expression of a phosphodiesterase *in trans* markedly reduced Clin110 attachment (Figure S4). Second, we analyzed strain SCV20265, a clinical CF isolate whose SCV morphology is abolished by transposon insertions in *yfiN* or *yfiB* [14]. Sequence analysis of the *yfiBNR* operon from SCV20265 revealed two *yfiN* mutations; the activating mutation G173D (Figure 2; Table 1) and an in-frame deletion of codons 255–257 within the GGDEF diguanylate cyclase domain (Table S3). To determine their individual contributions, *yfiBNR* operons containing one or both *yfiN* mutations present were introduced into the *att*::Tn*7* site of a Δ*yfiBNR* strain. G173D alone induced a robust SCV morphology in PA01, while the Δ255–257 deletion abolished the SCV phenotype in combination with G173D (Figure 7C). Furthermore, YfiN alleles containing only the Δ255–257 deletion were completely inactive on the basis of attachment assays with *yfiN* expression *in trans* (data not shown). While an SCV-inducing mutation was found in SCV20265 YfiN, the final protein is inactive. Clearly, additional SCV-inducing mutations must have arisen to complement the loss of YfiN activity, the nature of which is the subject of active investigation.

Together these data argue that the YfiBNR system is under both positive and negative selection in *P. aeruginosa* colonizing the lung of CF patients. Since SCV isolates have high reproductive costs [11], it is possible that alternating selection for rapid growth and persistence acts on the c-di-GMP network, thereby accumulating gain- and loss-of-function alleles in key components like YfiN.

### Yfi defines a widespread and highly modular bacterial signaling system

**Figure 8:**
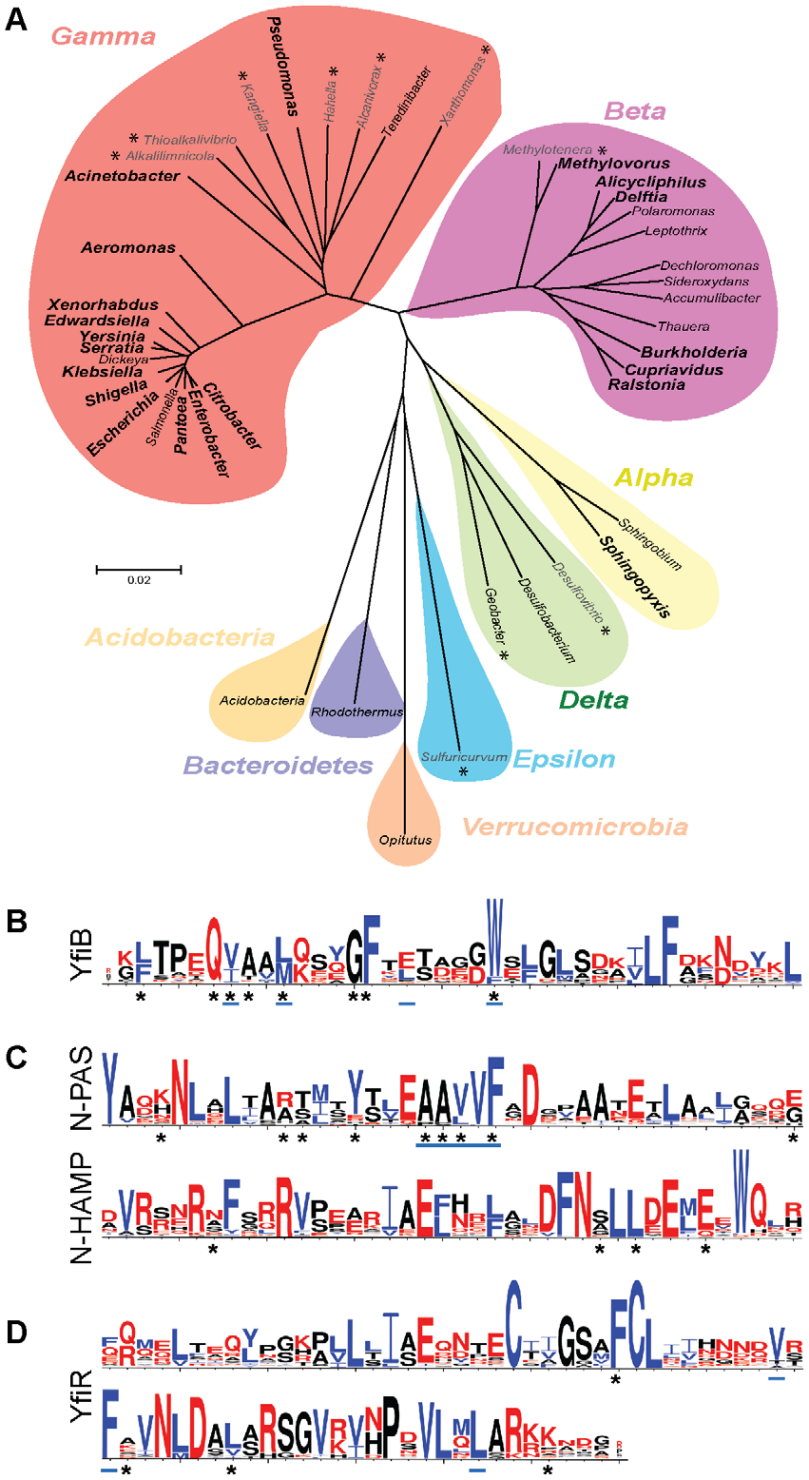
*In silico* analysis of the YfiBNR system. A) Lineage tree illustrating the distribution of the *yfiBNR* genes along bacterial lineages. Individual branches indicate separate genera. Genera marked in large, bold type contain species with complete *yfiBNR* operons. Those marked with an asterisk (*) contain species with *yfiB* and *yfiN* only, conserved and in synteny. The remaining genera contain species with two *yfi* genes conserved and in synteny (usually *yfiN* and *yfiR*) and a third *yfi* homolog elsewhere in the genome. Bacterial classes are indicated with coloured shading. Alpha, Beta etc. refer to the respective proteobacterial class. B-D) Weblogo representations of YfiBNR residue conservation. The height of the letter in each case indicates the degree of conservation at that position. Hydrophobic residues are coloured blue, hydrophilic residues red. Asterisks (*) indicate the sites of activating mutations, while blue underlining indicates those residues suggested to contribute to a hydrophobic protein binding site. B) YfiB residues 33–72. C) YfiN PAS-domain residues 48–87, YfiN HAMP-domain residues 198–237. D) YfiR residues 122–191.

Homologs of the YfiB, YfiN, and YfiR proteins were determined and plotted on a 16S rRNA-based phylogenetic tree to represent the taxonomic spread of the system (Figure 8A), for more details see Materials and Methods. 144 genomes were found to contain complete or partial *yfiBNR* operons. Genera containing complete, conserved *yfiBNR* operons (total 99) were found in the alpha-, beta- and gamma-proteobacteria, with most examples clustering in the gamma and beta classes. Two types of degenerate *yfi* operons were also identified. Firstly, operons containing *yfiN* and *yfiB* homologs in synteny, but no *yfiR*, were found in ten predominantly gamma-proteobacterial genomes (Table S4). In most cases, the transmembrane helices and GGDEF output domain of YfiN, as well as the PAL domain of YfiB were conserved. However, no homology was observed for the periplasmic domain of YfiN, and none of the proposed YfiR binding residues were conserved (Figure 8C, D), indicating that these systems function in a markedly different manner to *P. aeruginosa* YfiBNR. Secondly, 35 genomes were identified containing all three *yfi* homologs, but with only two of them in synteny (Table S4). Closer examination suggested that the genes in synteny were almost always *yfiN* and *yfiR*. The γ-proteobacteria *Salmonella* and *Dickeya* contain fully conserved *yfiR* and *yfiN* genes but lack *yfiB*. Interestingly, with growing evolutionary distance from *Pseudomonas* sp., the general divergence of the Yfi systems increased. Almost all of these operons lacked an *yfiB* homolog, and in many cases the GGDEF output domain of YfiN was replaced with GGDEF-EAL pairs, histidine-phosphotransfer domains (HPT) or sensor histidine kinases (Table S4). Variation was also seen at the level of signal input. In *Thauera* sp. MZ1T a system was identified with two YfiR homologs and a single YfiN with a hybrid sensor kinase output. The genomic location of the *yfiN-yfiR* homologs was highly variable, with some systems part of operons and others found alone. Nonetheless, alignment of PA01 YfiR and YfiN with some of the most divergent homologs revealed that the hydrophobic residues identified above as important for function were conserved throughout (Figure S5), suggesting that the basic concept of YfiR regulation of YfiN remains unchanged even in these highly divergent systems.

Altogether, these analyses suggest that the YfiR-YfiN[PAS] core represents a widespread, highly modular and versatile signaling system in bacteria that during evolution has integrated additional signal inputs through the acquisition of YfiB and distinct activities through the combination of different output domains.

## Discussion

During long-term colonization of CF lungs, *Pseudomonas aeruginosa* clones undergo specific genotypic adaptation to the host environment. As a result, the genotypes of strains present in advanced CF infections differ substantially from strains that originally invade the respiratory tract of these patients [2]. During adaptation to chronic behavior *P. aeruginosa* experiences a widespread loss of virulence factors and concomitant ‘induction’ of persistence traits. However, little is known about the exact nature of these traits or about the adaptive processes that lead to their expression. In this study we investigate specific genetic adaptations of the *P. aeruginosa* c-di-GMP signaling network and their role in the establishment of chronic infections of CF lungs. We demonstrate that during long-term lung infections, mutations affecting the activity of key regulators of c-di-GMP production invade the bacterial population in the lung, driving the formation of persistent SCV morphotypes *in vivo*. Our studies concentrate on the YfiBNR system, one of the previously identified clinically relevant systems for c-di-GMP production in *P. aeruginosa* [11]. The finding that the YfiBNR proteins are subject to both positive and negative selection allows us to make specific predictions regarding the adaptive processes occurring during long-term chronic infections of CF lungs, i.e. that the fitness landscape of the lung is heterogeneous and dynamic, providing selective pressure for both smooth and SCV phenotypes in different regions of the lung, or periods during infection. The recognition of genetic changes occurring in the patient was facilitated by a comprehensive analysis of Yfi-mediated regulation in *P. aeruginosa*, emphasizing the importance of a thorough molecular and mechanistic understanding of physiological and regulatory processes for the analysis of adaptive behavior *in vivo*.

While the epistasis and physiological significance of the YfiBNR system has been described previously [11], [35], its mechanism of action has remained largely unknown. We provide biochemical and genetic evidence that the activity of membrane-integral diguanylate cyclase YfiN is suppressed via interaction between its extracellular PAS domain and the periplasmic repressor protein YfiR. Release of repression, either by YfiR disruption or by YfiB-mediated sequestration to the outer layer of the cell envelope leads to a conformational shift in YfiN that propagates through the PAS and transmembrane domains to switch the cytoplasmic HAMP domain from an inactive to an active conformation. This activating conformational change is subsequently transmitted to the C-terminus, where DGC activity is likely determined by the formation of a properly aligned GGDEF homodimer [53], [66], [67]. Thus, YfiN appears to function by switching between discrete inactive and active functional states depending on the presence or absence of bound YfiR. In this respect, YfiN is similar to a number of other well-characterized transmembrane signaling proteins, including histidine kinases and methyl-accepting chemotaxis proteins [56] in which ligand binding provokes activating conformational changes that propagate throughout the protein structure.

Our analyses place YfiR at the center of the Yfi signaling pathway with two potential interaction partners, YfiN and YfiB, localized in the inner and outer membrane, respectively. Specific partner selection of YfiR would then determine YfiN signaling activity. YfiR appears to be unique, without functional homologs in other signaling systems. Nonetheless, the combination of mutagenesis, structural modeling and analysis of sequence conservation applied in this study allowed us to propose that YfiR may bind to YfiN via a hydrophobic binding site composed of residues in the C-terminal region of the protein. Sequence conservation indicates that hydrophobic amino acids (Val160, Phe162, Leu183) on the surface of the modeled YfiR structure may be especially important in this regard. Consistent with such a mode of action, compensatory mutations in YfiR that are able to suppress activated forms of YfiN are positioned in the immediate vicinity of this hydrophobic patch. Likewise, our genetic studies indicate that YfiR binds to a highly conserved stretch of five amino acids (AAVVF) on the surface of the YfiN PAS domain. Even conservative substitutions in this region abolish the YfiR binding surface and lead to YfiN activation. It is possible that the valine and alanine residues coordinate the central phenylalanine to protrude into the periplasm thereby providing a hydrophobic binding site for YfiR. To our knowledge, this is the first recorded example of a prokaryotic PAS domain that signals via protein-protein interaction. Structural studies are underway to clarify the molecular details of this interaction.

YfiB spans the peptidoglycan and the outer membrane and appears to function by sequestering YfiR away from the inner membrane and YfiN. While direct YfiR-YfiB interaction could not be demonstrated, YfiR localization to the outer membrane fraction requires the presence and activity of YfiB. The location of activating mutants in YfiB provided insight into the mechanism of YfiB action, leading us to propose that activation may result from the exposure of a patch of hydrophobic residues on the YfiB surface, particularly the highly conserved tryptophan at position 55 (Figure 8). According to our model of YfiB structure and topology, these hydrophobic residues form an YfiR binding site immediately proximal to the insertion site of the protein into the outer membrane. Since both peptidoglycan binding and outer membrane anchoring are essential for YfiB function, the protein might sense envelope integrity or the distance between the two outermost layers of the cell, a mechanism that has also been suggested for the outer membrane protein Pal [51]. Changes in the relationship between the outer membrane and peptidoglycan would expose or obscure the hydrophobic YfiR binding site, controlling the amount of YfiR bound to YfiB and hence the level of YfiN activation. Such a model is supported by the observation that shortening of the linker between the Pal-like domain of YfiB and its anchoring site in the outer membrane leads to YfiR recruitment and activation of YfiN, while a longer linker retains overall YfiB activity but does not lead to enhanced activation. A corollary of this model is that the YfiR-YfiN interaction would need to exist in a state of dynamic equilibrium, with some unbound YfiR and YfiN present at all times. Rather than directly challenging the YfiR-YfiN interaction, YfiB would act as a ‘sink’ for unbound YfiR, removing it from the periplasm and thus shifting the equilibrium towards unbound, active YfiN.

The model for YfiBNR function proposed above implies a biological role for the Yfi system in sensing physical stresses affecting the outer membrane and/or the peptidoglycan, and triggering c-di-GMP-dependent outputs as a response to that stress. The nature of these output systems is the subject of ongoing research, but is currently thought to focus on the control of exopolysaccharide production at the transcriptional and post transcriptional levels [11]. Our experiments failed to directly demonstrate a role for YfiB in the response to envelope stress. Although the Yfi system as a whole is required for full biofilm induction at high osmolarity or in the presence of SDS, two stressors that are known to affect the integrity of the cell envelope, we could not demonstrate a specific role for YfiB in this process. In addition to YfiB-mediated activation, YfiN can be activated by YfiR disruption. This is most visible in a genetic *yfiR* mutant, but may also occur when YfiR does not fold correctly due to incorrect/incomplete cysteine crosslink formation. The four cysteine residues in YfiR are strictly conserved amongst complete YfiBNR systems, supporting their importance for YfiR function. In response to external reducing agents *P. aeruginosa* stages a biofilm response that fully depends on the presence of YfiN and YfiR, but not YfiB, and which greatly enhances PA01 tolerance under these conditions in comparison to an *yfiBNR* mutant strain. YfiR misfolding thus potentially represents a second mode of YfiBNR sensory input, and suggests a role for the system in the response to reducing environments. For example, it is conceivable that the Yfi system is able to sense anoxic conditions through the redox sensitive cysteines of YfiR and by that induce biofilm in the absence of oxygen. The option to employ YfiR directly as a signal input domain, either as a cysteine-dependent redox sensor or through another as-yet-undiscovered mechanism, predicts the existence of Yfi-like signaling systems in which YfiB is missing. This is indeed the case. While complete *yfiBNR* systems are widespread in the gamma- and beta-proteobacteria (with the alpha-proteobacteria *Sphingopyxis* representing the only genus external to these two classes) many YfiNR pairs lacking an YfiB component are found in species both closely related and widely divergent from *P. aeruginosa*. In particular, species in the *Salmonella* and *Dickeya* genera have conserved YfiN and YfiR homologs, but lack YfiB. Given the positions of *Salmonella* and *Dickeya* in the YfiBNR lineage tree it seems likely that these genera may once have had functional YfiB homologs, but have since lost them. Evolution has not only modulated the input side of the YfiBNR system, the output domains of more divergent Yfi homologs are also variable. Careful *in silico* analysis identified histidine kinases, GGDEF/EAL pairs and MCP domains in place of the YfiN GGDEF domain. Despite this diversity of signaling outputs, the residues predicted for YfiN-YfiR interaction are well conserved. This led us to hypothesize that this YfiR-YfiN [PAS/HAMP] core may be a more ancient signaling module, which in evolution has been connected in a modular fashion to different output and additional input domains. This core module, characterized by the binding of a periplasmic repressor to the PAS-like domain of a transmembrane signal transduction system, represents a novel and apparently widespread signaling motif in bacteria. The YfiN-YfiR interaction shares some striking characteristics with the LapD-LapG system of *Pseudomonas fluorescens* [68], [69], [70] and *Pseudomonas putida* [71]. The unique periplasmic domain of the c-di-GMP signaling protein LapD sequesters the periplasmic protease LapG when c-di-GMP is present, preventing it from cleaving the adhesin LapA. Periplasmic protein interaction systems such as LapAG and YfiBNR may well be representatives of a broader regulatory principle in bacterial signaling.

The mutational analysis described in this study demonstrated several possible genetic routes to *yfi*-mediated SCV formation. In addition to loss-of-function mutations in *yfiR*, mutations that mimic an active conformation may arise at specific locations in the PAS, HAMP or transmembrane regions of YfiN, or towards the N-terminus of YfiB. When analyzing the *yfi* operons of clinical SCVs we identified several that contained either an inactivating mutation in *yfiR* or an activating mutation in *yfiN*, arguing that the Yfi signal transduction system is under strong positive selection in the lung environment. Apparently, one route to generate SCVs *in vivo* is by an increase of c-di-GMP through mutations that activate or de-repress a diguanylate cyclase. Consistent with this, the methylesterase *wspF*, whose disruption induces c-di-GMP dependent SCV formation *in vitro* [72], was shown to be mutated in a *P. aeruginosa* strain following adaptation to the CF lung [2]. Similarly, Rau et al. have identified a persistent CF isolate in which the GGDEF/EAL protein MorA is strongly upregulated [73]. Furthermore, we find that in a large fraction of *P. aeruginosa* SCVs isolated from CF lungs the characteristic morphotype can be alleviated or reversed to a more rapidly growing smooth phenotype by the expression of a phosphodiesterase (Jaeger, Bos and Jenal, unpublished). Of the five SCV-inducing mutations isolated in the *yfi* system, four were gain of function mutations in YfiN, with only one loss-of-function mutation found in YfiR. This is surprising, as one expects loss-of-function mutations in *yfiR* to occur more frequently than activating mutations in *yfiN*. These findings seem to suggest that the strongest Yfi-mediated SCVs, produced when YfiR is absent and YfiN is fully active, are disfavored in the CF lung compared with weaker morphotypes produced by activated YfiN alleles. It is possible that YfiR has a supplementary role in cell signaling and that loss-of-function mutations in the *yfiR* gene incur an unforeseen fitness cost in the human host. Alternatively, activating mutations in *yfiN* might be favored because they allow the system to retain some degree of flexibility, where a secondary genetic or regulatory increase in YfiR activity could override the activating effect of the original mutation in *yfiN*. Careful analysis of selected clinical SCVs indeed provided evidence for consecutive rounds of mutations affecting the *yfi* system. The finding that some clinical strains contained both gain- and loss-of function mutations in *yfiN* strongly suggested that SCVs can function as an environmental pool for the generation of new smooth morphotypes. Apparently, SCVs do not represent a dead end in the dynamic and heterogeneous environment of chronically infected CF airways and may be continuously favored and disfavored over time and/or different lung environments. In this way the behavior of clinical *P. aeruginosa* SCVs reflects that of *P. fluorescens* cultured *in vitro* [36], where sequential gain and loss of function mutations arose in c-di-GMP related systems (including Yfi/Aws), in response to environmental changes. The variable fitness of SCVs might well be explained by the characteristic slow growth of these morphotypes [11], which may contribute substantially to persistence under very stringent *in vivo* conditions but pose dramatic costs under conditions that favor rapid growth. E.g. antibiotic treatment regimens or other insults that alter the fragile homeostasis between airway pathogens and local host defenses [74] might favor selection for persistence traits, while in the absence of such conditions rapidly growing sub-types might have a considerable advantage. Continuous adaptation of the c-di-GMP network could facilitate these physiological adjustments and thereby contribute to the *in vivo* fitness of *P. aeruginosa* during chronic lung infections.

CF patients are permanently colonized by *P. aeruginosa* with the same bacterial lineage persisting continuously in the lungs for years or even decades and without being eradicated by chemotherapy. The development of effective treatments rests on a detailed understanding of how bacteria adapt to the airway and resist host defenses and antibiotics. Several reports have indicated a striking correlation between mutagenesis and persistence of *P. aeruginosa* in CF lungs [2], [75], [76], [77]. Smith et al. [2] have reported that during chronic infection *P. aeruginosa* adapts by loss-of-function mutations that remove virulence factors required for the acute phase of infection. But while this study outlines factors that are disadvantageous in chronic infection, it does not provide information about what is needed to enhance fitness in CF airways and sustain the clonal expansion of *P. aeruginosa* during chronic infection. Alleles causing (often subtle) positive changes in protein function are more difficult to identify and rationalize than loss-of-function mutations and often require experimental input to be validated. E.g., the identification and elucidation of the *yfi* gain-of-function alleles in clinical isolates was critically dependent on the mechanistic understanding that could only be provided by a thorough molecular analysis of the pathway. Based on these studies we propose that rapid evolution of diguanylate cyclases and possibly other components of the c-di-GMP network contributes to the remarkable flexibility and degree of persistence of *P. aeruginosa* cells in CF lungs. A thorough understanding of the underlying physiological processes is mandatory to predict, delineate and interfere with these *in vivo* selection processes.

## Materials and Methods

### Ethics statement

The clinical *Pseudomonas aeruginosa* strains used for this research were cultured from patient samples collected for routine microbiological testing at the University Children's Hospital, Basel or the Medical Microbiological Department of the Hannover Medical School. Subculturing and analysis of bacteria was performed anonymously. No additional procedures were carried out on the patients. Cultures were sampled following regular procedures with written informed consent, in agreement with the guidelines of the “Ethikkommission beider Basel EKBB” or with approval by the Ethical Board of the Hannover Medical School.

### Strains and growth conditions

Strains and plasmids used in this study are listed in Table S1. Primers are listed in Table S2. Unless otherwise stated, all *P. aeruginosa* and *E. coli* strains were grown at 37°C in Luria Bertani (LB) medium [78], solidified with 1.3% agar where appropriate. For *P. aeruginosa*, gentamycin was used at 30 µg/ml (*E. coli* 20 µg/ml), carbenicillin at 100 µg/ml and tetracycline at 50/100 µg/ml (*E. coli* 12.5 µg/ml). For *E. coli*, ampicillin was used at 100 µg/ml. Congo Red dye was added to a final concentration 0.04%. For inducible plasmids, IPTG was added to a final concentration 0.2 or 1 mM and arabinose to 0.2% as appropriate.

### Molecular biology procedures

Cloning was carried out in accordance with standard molecular biology techniques. To produce a chromosomal flag-tagged copy of *yfiN E. coli* DY330 [79] was transformed with plasmid pMR20-*yfiBNR* [11]. The PCR fragment amplified with primers A and B from plasmid pSUB11 [80] were then used to produce a C-terminal *yfiN* fusion in pMR20 by the method described by Yu et al. [79]. The resulting *yfiN-flag* fragment was then ligated between the *Hin*dIII and *Bam*HI sites of pUC18T-mini-Tn7T-Gm [81].

The *yfiR* screening plasmid pMR*-yfiR-flag* was constructed by replacement of the *lac-promoter*/*lacI* fragment of pMR20*-yfiR-M2* [11] with a chloramphenicol cassette amplified using primers C and D, according to the method of Yu et al. [79]. The chloramphenicol gene was then excised by Flp-mediated excision using the plasmid pFLP2 [82]. To produce the *yfiN* screening plasmid pGm*-yfiprom-N*, the tetracycline resistance cassette was excised from pME6032 [83] between *Bam*HI and *Xba*I, and replaced with the *aacCI* gentamycin resistance cassette, amplified with primers E and F from pBBR-MCS5 [84]. The *lac-promoter*/*lacI* fragment was then excised between *Eco*RI and *Bam*HI and the *Eco*RI site was blunted with klenow polymerase. The *yfiN* fragment was amplified with primers G and H from PA01 Δ*yfiR* [11] and ligated between *Bam*HI and the blunt site.

The three pME-*yfiB-PG-* variants were constructed by ligation of *yfiB* fragments (amplified with primers I and U, from the appropriate pME6032-*yfiB* gain-of-function plasmids) between the *Eco*RI and *Asc*I sites of pME6032-*yfiB* [11]. To make the *yfiB*-LA- constructs, *yfiR* was amplified with primers V and P, and *yfiB* with primers W and J from genomic DNA (or pME-*yfiB-PG-* as appropriate). The *Mfe*I-*Pst*I *yfiR* fragment and the *Pst*I-*Bam*HI *yfiB* fragments were then ligated between the *Eco*RI and *Bgl*II sites of pME6032. The long/short *yfiB* inserts were produced by SOE-PCR using primers AH/AI together with primers X/Y or Z/AA, and ligated into pME6032 between *Eco*RI and *Bgl*II. To produce pME-araC-*dsbA*, the *dsbA* fragment amplified with primers K and L from PA01 genomic DNA was ligated between the *Eco*RI-*Kpn*I sites of pME-araC [11], [83]. To make pME-araC-*yfiR* the PCR product amplified with primers V and P was cut with *Mfe*I-*Bam*HI and ligated between the *Eco*RI-*Bgl*II sites of pME-araC.

To produce pTn*7-yfiBN20265-R*, the *yfiBNR* operon of SCV20265 was amplified using primers G and J and ligated between the *Hind*III and *Bam*HI sites of pUC18T-mini-Tn7T-Gm. The G173D and Δ255-7 variants were produced by digesting *yfiBNR* PCR products from SCV20265 and WT PA01 templates at a *Pst*I site midway between the two mutations, then ligating both inserts between *Bam*HI and *Hind*III as above. Clinical *yfiN*/*yfiR* mutant alleles were produced by SOE PCR using primers AR/AS and AJ-AQ for yfiN, and AZ/BA and AT-AY for *yfiR*, respectively. Inserts were then ligated into *Nco*I-*Bam*HI-cut pME6032*-Gm* or *Hind*III-*Bam*HI-cut pMR*-yfiR-flag* respectively.

### XL1-red mutagenesis

Mutator strain *E. coli* XL1-red (Stratagene) was transformed with the plasmid pGm*-yfiprom-N* according to the method of Chung et al. [85] and plated onto LB gentamycin plates. After overnight growth at 37°C the transformant colonies were pooled and plasmid DNA extracted. This pooled DNA was then used to transform the screening strain Δ*yfiNR* pMR*-yfiR-flag*. Samples were plated onto LB plates containing gentamycin, tetracycline and Congo Red and transformants exhibiting an SCV phenotype were selected as potentially carrying activated YfiN alleles. These SCVs were restreaked onto fresh plates and the *yfiN* gene amplified in each case via colony PCR, using primers G and H. Mutations in *yfiN* were then identified by sequencing.

### Screening by error-prone PCR

Plasmids pME6032-*yfiB* [11] and pMR*-yfiR-flag* were used as templates for error-prone PCR reactions following the method described in [86], and consisting of 2 min 94°C, then 25 cycles of 45 sec 94°C, 45 sec 55°C and 90 sec 72°C, before a final extension for 10 min at 72°C. Each 50 µl reaction contained 0.2 mM dNTPs, Taq polymerase buffer, 5U Taq polymerase, 0.3 µM forward and reverse primers (I/J and G/P), 1.0 µl template DNA and 0.5–1.0 µl mutagenesis buffer (4 mM dTTP, 4 mM dCTP, 27.5 mM MgCl_2_, and 2.5 mM MnCl_2_). Pools of mutated *yfiR* and *yfiB* fragments were then ligated into their parent vectors between *Hin*dIII - *Bam*HI, and *Eco*RI - *Bgl*II respectively. To screen for *yfiB* mutants, Δ*yfiBNR* Tn*7*::*yfiNR* [11] was transformed with pooled pME6032-*yfiB* and SCV transformants were isolated from selective LB plates. Mutations in *yfiB* were identified by sequencing. For *yfiR* mutants, Δ*yfiNR* was transformed with pooled pMR*-yfiR-flag* and the resulting transformant colonies used to make electrocompetent cells. Plasmids carrying the 20 activated *yfiN* alleles were extracted and purified, then used to transform the Δ*yfiNR* pMR*-yfiR-flag* pooled cells. Transformants were screened on selective LB plates for smooth colonies containing potential dominant YfiR alleles. To eliminate suppressor mutations in the screening strain, pMR*-yfiR-flag* DNA was extracted from smooth strains and used to transform Δ*yfiNR* containing the relevant activated-*yfiN* plasmid. The *yfiR* alleles present in those strains that displayed a smooth morphotype at this stage were analyzed by sequencing.

### Transduction with phage E79tv2

The E79tv2 [87] transducing lysate for the Tn*5*::*wspR* strain was prepared and used according to the protocol described in our previous work [11].

### Deletion of the *dsbA* gene

The strains PA01 Δ*dsbA* and Δ*yfiBNR* Δ*dsbA* were constructed via an adaptation of the protocol described elsewhere [88]. Briefly, the *dsbA* deletion construct was produced by SOE-PCR using primers Q-T, and contained homologous flanking regions to the target gene. This construct was ligated into pME3087 between *Hind*III-*Bam*HI. The resulting vector was then used to delete *dsbA* by two-step allelic exchange. Following transformation into the target strain, single crossovers were selected on tetracycline and restreaked. Cultures from single crossovers were grown overnight in LB medium, and diluted 1∶100 into fresh medium. After 2 hours, 20 µg/ml tetracycline was added to inhibit the growth of cells that had lost the tetracycline cassette. After a further hour of growth, 2000 µg/ml carbenicillin was added to select against growing bacteria. Cultures were grown for a further 4–6 hours, before cells were harvested by centrifugation, washed once in LB and used to inoculate an overnight culture. This counter-selection was done twice, before plating of a dilution series of the final samples onto LB agar. Individual colonies were patched onto LB plates ± tetracycline, tetracycline sensitive colonies were tested for deletion of *dsbA* by colony PCR.

### Attachment assays

Assays to test the different alleles of the *yfiBNR* system were performed as described in [11], [89]. Briefly, 96 well plates containing 150/200 µl LB medium/well were inoculated with single colonies using sterile toothpicks, and incubated overnight at 37°C without shaking. Plates were washed three times with distilled water. The attached cell material was then stained with 0.1% Crystal Violet solution (5% methanol, 5% isopropanol) before further washing to remove excess dye. Crystal Violet was re-dissolved in 20% acetic acid solution and absorbance measured at 600 nm. Assays were performed with 6 wells/strain and repeated independently for each experiment.

The attachment assays for phenotypic testing of stress conditions were performed with the following modifications. Strains were grown on LB plates O/N at 37°C then scraped from the plates and diluted in the appropriate medium used for the experiment. 96 well plates containing 200 µl medium/well were inoculated with the diluted bacterial suspension to a final OD_600_ of 0.001, plates were incubated overnight at 37°C without shaking. Envelope stress was tested on M9 minimal medium (47.6 mM Na_2_HPO_4_, 22 mM KH_2_PO_4_, 8.6 mM NaCl, 18.6 mM NH_4_Cl, 2 mM MgSO_4_, 0.1 mM CaCl_2_, 0.03 mM Fe-III-citrate) containing 10 mM Na-succinate as a carbon and energy source and 0.01–0.25% sodium dodecyl sulfate (SDS) [90] and with osmotic stress minimal medium M63 (100 mM KH_2_PO_4_, 15 mM (NH_4_)_2_SO_4_, 2 µM FeSO_4_, 1 mM MgSO_4_, 0.001 mg/ml Thiamin) [78] with 20 mM Na-succinate, 0.2% Glucose and 0.01–0.4 M NaCl [91]. Reducing conditions were tested on MHB (Mueller-Hinton broth, cation adjusted, Becton Dickinson) containing 1.25–20 mM Dithiothreitol (DTT). Assays were performed at least 3 times independently with 12 wells/condition tested, shown is the mean of all experiments.

### Immunoblot analysis

Samples were separated on 15% Tris-HCl gels and blotted onto polyvinylidene difluoride (PVDF) membranes (Millipore). Membranes were incubated overnight in blocking solution (1× PBS pH 7.4, 0.01% Tween20, 5% milk powder), after which proteins were detected with 1/5000 (α-M2, α-YfiB) or 1/2000 (α-YfiN) specific antiserum and 1/10,000 rabbit anti-mouse (α-M2) or swine anti-rabbit (α-YfiB, α-YfiN) secondary antibody (DakoCytomation). Bound antibodies were visualized using ECL chemiluminescent detection reagent (Perkin-Elmer). Samples were normalized following comparison of optical density for the initial cell samples in each case.

### Co-immunoprecipitation

PA01 overnight cultures (or colonies scraped from agar plates, in the case of SCV strains) were pelleted by centrifugation and re-suspended in ice-cold IP buffer (20 mM HEPES pH 7.4, 100 mM NaCl, 1 mM EDTA, 1.0% ^v^/_v_ Triton X-100, protease inhibitor), then incubated at 4°C with end-over-end agitation for 6 hours. All subsequent steps were carried out on ice. Samples were centrifuged (15,000 g, 20 min, 4°C), and the supernatant was removed and incubated with 20 µg/ml protein-A agarose beads at 4°C with end-over-end agitation for 30 min to remove non-specifically binding proteins. The samples were then centrifuged (3,000 g, 1 min 4°C) to pellet the beads, an aliquot of the supernatant was taken for analysis, and the remaining supernatant was incubated overnight with 20 µg/ml ANTI-flag M2 affinity gel (Sigma) at 4°C with end-over-end agitation. Samples were pelleted by centrifugation (3,000 g, 1 min 4°C), the supernatant was discarded and the beads re-suspended in 1.0 ml ice-cold IP buffer. This wash step was repeated between 3 and 5 times. The beads were then re-suspended in SDS sample buffer, boiled for 10 minutes at 95°C and pelleted by centrifugation. Interacting proteins in the supernatant were detected by immunoblotting.

### Membrane fractionation

The fractionation protocol was adapted from the method described in [92]. 50 ml overnight cultures of PA01 strains were pelleted by centrifugation, washed once with Tris buffer (30 mM Tris pH 8.0), and re-suspended in 6.0 ml Tris buffer containing 20% ^w^/_v_ sucrose, 1 mg DNase, 1 mg RNase, and 2 mg lysozyme. Samples were incubated for 20 minutes at room temperature, then lysed by French Press, diluted to 10% sucrose with Tris buffer and centrifuged to remove cell debris (3,500 g, 15 min, 4°C). Protease inhibitors were added at this stage as a precaution. The soluble and membrane fractions were then separated by ultracentrifugation (160,000 g, 105 min, 4°C) on a Tris buffered 15%/70% sucrose gradient. The membrane fraction was recovered from the 15%–70% interface and diluted to about 30% with Tris buffer, before loading onto a Tris buffered, sucrose step-gradient (steps contained 70, 64, 58 and 52% sucrose). The samples were then ultracentrifuged for 18 hours (160,000 g, 4°C) to separate inner and outer membranes. The four bands visible after ultracentrifugation were recovered, diluted in 1–2 volumes Tris buffer and pelleted (15,000 g, 35 min, 4°C), before being re-suspended in Tris buffer and analyzed by immunoblotting. Western blot samples were normalized according to the total protein content of the different fractions, determined with a Protein assay kit (BioRad).

### Quantitation of cyclic-di-GMP levels

C-di-GMP levels of clinical strains were measured by liquid chromatography-tandem mass spectrometry after [93]. The protein content of each pellet (post-extraction) was determined with a Protein Assay kit (BioRad). Measurements were in triplicate and values expressed as pmol c-di-GMP/mg protein.

### Sequence analysis of clinical SCV strains

In total 45 auto-aggregative SCV strains isolated from the sputum of CF patients were grouped in three pools of 15 strains. Each strain was cultivated individually in 20 ml LB medium at 37°C and shaking at 180 rpm to an OD_600_ of 1.0. 1 ml of each culture was harvested by centrifugation (8000 rpm, 1 minute, 4°C) and the cell pellet was washed once with 1 ml sterile deionized water. Pellets were resuspended in sterile deionized water, pooled at equal amounts and DNA was isolated from the pooled cell suspensions using the DNeasy Blood & Tissue Kit (Qiagen) according to the manufacturer's instructions.

DNA samples were further prepared and sequenced using 76 bp paired end sequencing on a Genome Analyzer II-x (Illumina). Libraries of 250 bp prepared according the manufacturer's instructions “Preparing Samples for Paired-End-Sequencing”. Cluster generation was performed using the Illumina cluster station, sequencing for read 1 and read 2 on the Genome Analyzer followed a standard protocol. The fluorescent images were processed to sequences using the Genome Analyzer Pipeline Analysis software 1.6 (Illumina).

The sequence reads were quality-trimmed using the perl script Trim.pl by Nik Joshi (obtained from http://wiki.bioinformatics.ucdavis.edu/index.php/Trim.pl) in the “windowed adaptive trimming” mode, removing any sequence with a quality score <10. Detection of single nucleotide polymorphisms (SNPs) was performed with the MAQ software [Li et al, 2008, Genome Res 18∶1851] using a perl script that calculates the frequencies of SNPs in pooled sequences [Holt et al., 2009, Bioinformatics 25∶2074].

### Identification of YfiBNR homologs in other systems

An initial set of genomes displaying synteny conservation among homologs to *PA1119* (GenBank:15596316), *PA1120* (GenBank:15596317) and *PA1121* (GenBank:15596318) from *Pseudomonas aeruginosa* PAO1 (GenBank:AE004091) was manually defined. Synteny conservation was assessed for complete microbial genomes of the RefSeq database on the MAGE database server [94]. The three sets of protein sequences corresponding to each homologous group were aligned with CLUSTAL W (default settings) [95]. Hidden Markov Models were then computed with HMMER.3 (http://hmmer.org/). Models were calibrated and searched against a local copy of the microbial complete genome database (NCBI) with HMMER.3. A series of Perl scripts were used to sort the outputs and to count occurrences of complete or partial systems as described in Figure 8. Occurrences of homologs (E-value<10^−4^) were then reported on an illustrative phylogenetic tree based on 16S rRNA sequences from the ribosomal database project (RDP, http://rdp.cme.msu.edu/index.jsp) [96]. All sequences were >1200 nucleotides and tagged as “good quality” according to RDP. Type strains and isolated samples were preferred. At least two sequences/genus were downloaded as alignment files from RDP. Consensus was inferred using EMBOSS (http://www.sanger.ac.uk/ Software/EMBOSS). Genus consensuses were aligned with CLUSTAL W (default settings) and phylogenetic analyses were conducted in MEGA4 [97]. Evolutionary history was inferred using the UPGMA method [98], and evolutionary distances were computed using the Maximum Composite Likelihood method. All positions containing gaps and missing data were eliminated (Complete deletion option), leaving a total of 1143 positions in the final dataset.

Molecular models of the YfiBNR proteins were produced using HHPRED (http://toolkit.tuebingen.mpg.de/hhpred) and visualized with PYMOL (http://pymol.org/). Models were based on the following structures: YfiN PAS: 1p0z_A, YfiN HAMP: 3lnr_A, YfiR: 2iss_D, 2ywj_A, 3lft_A, 3dfu_A, 2abw_A, 2ioj_A, and 1yqg_A, YfiB: 2kls_A and 2ldt_A. Sequence conservation plots were visualized using WebLogo 3 [99].

## Supporting Information

Figure S1 **Localization and stability of YfiR.** A) Outer Membrane localization of YfiR-flag. Immunoblot of fractionated membrane samples stained with M2 antisera. The panel shows the soluble and the outer membrane fractions for *ΔyfiBNR, ΔyfiNR* and *ΔyfiBNR* Tn*7*::*yfiNR* harboring pMR*-yfiR-flag* (p-*yfiR*). B) Stability of YfiR-flag in whole cell lysate. Immunoblot of ΔyfiR, ΔyfiNR and ΔyfiBNR with yfiR-flag inserted into the att-Tn7 site, and one time point with the strains over expressing YfiR (ΔyfiBNR, ΔyfiNR and ΔyfiBNR Tn7::yfiNR harboring pMR-yfiR-flag (p-yfiR)). ‘t’ indicates different time points (hours post inoculation); ‘O/N’ overnight incubation.
(PDF)

Figure S2 **Colony morphologies with activated YfiN alleles.** A) Colony morphologies of *ΔyfiNR* pGm*-yfiprom-N*, pMR*-yfiR-flag* strains with the point mutants indicated present in YfiN. ‘p*yfiR* p*yfiN*’ denotes the wild-type control; ‘p*yfiN*’ denotes a strain containing pGm*-yfiprom-N* only. B) Colony morphologies for cross-complementation strains. The YfiN (bold) and YfiR (italic) mutant alleles present are shown in each case.
(TIF)

Figure S3 **Attachment of Yfi mutants in response to the presence of SDS or changes in osmolarity.** A) Attachment on M9 medium with 20 mM Na-succinate and increasing concentrations of SDS is shown relative to PA01. B) Attachment with low and high osmolarity, on M63 medium with 20 mM Na-succinate, 0.2% glucose, and increasing concentrations of NaCl is shown relative to PA01. 50% and 75% indicate dilutions of the medium with distilled water. ΔBNR indicates Δ*yfiBNR* and ΔB indicates Δ*yfiBNR* Tn*7*::*yfiNR.* Bars represent the level of absolute attachment and curves represent optical density (OD) of total cells with standard errors.
(PDF)

Figure S4 **Attachment of clinical iosolates in the presence of a phosphodiesterase.** The effect of a PDE, in Clin110 and Clin163, on attachment is shown relative to strains expressing an active site mutant (PDE*). The PDE (PA5295) and the active site mutant are expressed from a vanillate inducible vector (pBV-PA5295 and pBV-PA5295_E328A_ respectively).
(PDF)

Figure S5 **Sequence conservation across distant YfiN/YfiR homologs.** Sequence alignments for YfiR (residues 55–190) and the YfiN PAS domain (residues 61–115), across PA01 and five distant homologs. The five species whose *yfiNR* genes were compared were: *Opitutus terrae* PB90-1, *Acidobacteria bacterium* Ellin345, *Geobacter* sp. M21, *Rhodothermus marinus* DSM 4252, and *Desulfobacterium autotrophicum* HRM2. Fully conserved residues are marked with an asterisk (*), similarities are marked with (:) or (.). Putative hydrophobic binding site residues are enclosed in black boxes. Residues are colored according to the chemical nature of their side chains.
(PDF)

Table S1 **Bacterial strains and plasmids used in this study.**
(PDF)

Table S2 **Primers used in this study.**
(PDF)

Table S3 **Single nucleotide polymorphism (SNP) positions in the sequences of clinical SCVs, compared to** ***P. aeruginosa*** **PA01.**
(PDF)

Table S4 **Identification of YfiBNR homologs in other systems.**
(PDF)
