# Supplementary figures and images for: The YfiBNR Signal Transduction Mechanism Reveals Novel Targets for the Evolution of Persistent Pseudomonas aeruginosa in Cystic Fibrosis Airways
Source: PLoS Pathog. 2012 Jun 14;8(6):e1002760. doi: 10.1371/journal.ppat.1002760 (PMC3375315; doi:10.1371/journal.ppat.1002760)

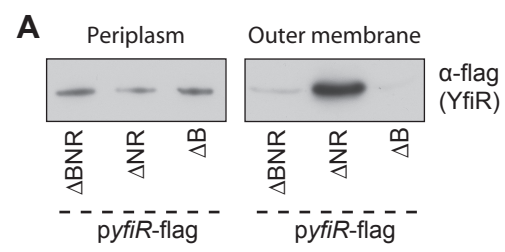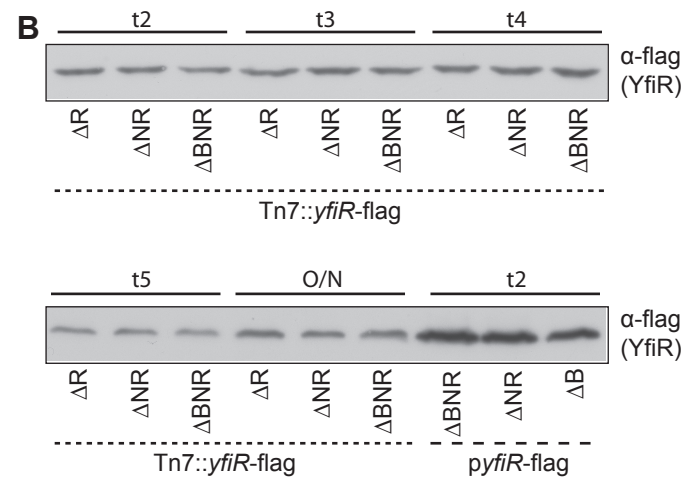

Supplement: Figure S1 — Localization and stability of YfiR. A) Outer Membrane localization of YfiR-flag. Immunoblot of fractionated membrane samples stained with M2 antisera. The panel shows the soluble and the outer membrane fractions for ΔyfiBNR, ΔyfiNR and ΔyfiBNR Tn7::yfiNR harboring pMR-yfiR-flag (p-yfiR). B) Stability of YfiR-flag in whole cell lysate. Immunoblot of ΔyfiR, ΔyfiNR and ΔyfiBNR with yfiR-flag inserted into the att-Tn7 site, and one time point with the strains over expressing YfiR (ΔyfiBNR, ΔyfiNR and ΔyfiBNR Tn7::yfiNR harboring pMR-yfiR-flag (p-yfiR)). ‘t’ indicates different time points (hours post inoculation); ‘O/N’ overnight incubation. (PDF) [file ppat.1002760.s001.pdf]

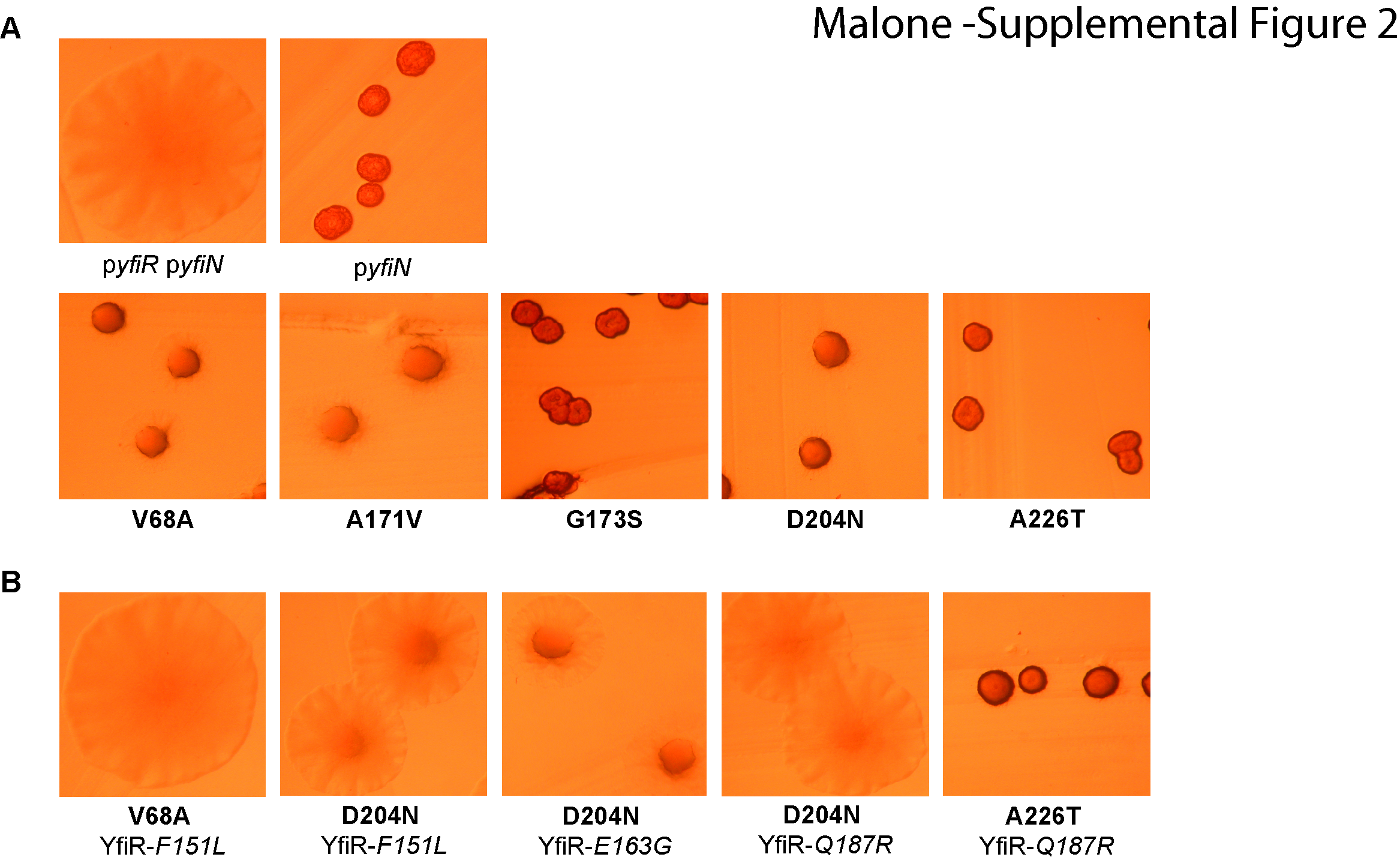

Supplement: Figure S2 — Colony morphologies with activated YfiN alleles. A) Colony morphologies of ΔyfiNR pGm-yfiprom-N, pMR-yfiR-flag strains with the point mutants indicated present in YfiN. ‘pyfiR pyfiN’ denotes the wild-type control; ‘pyfiN’ denotes a strain containing pGm-yfiprom-N only. B) Colony morphologies for cross-complementation strains. The YfiN (bold) and YfiR (italic) mutant alleles present are shown in each case. (TIF) [file ppat.1002760.s002.tif]

**A**

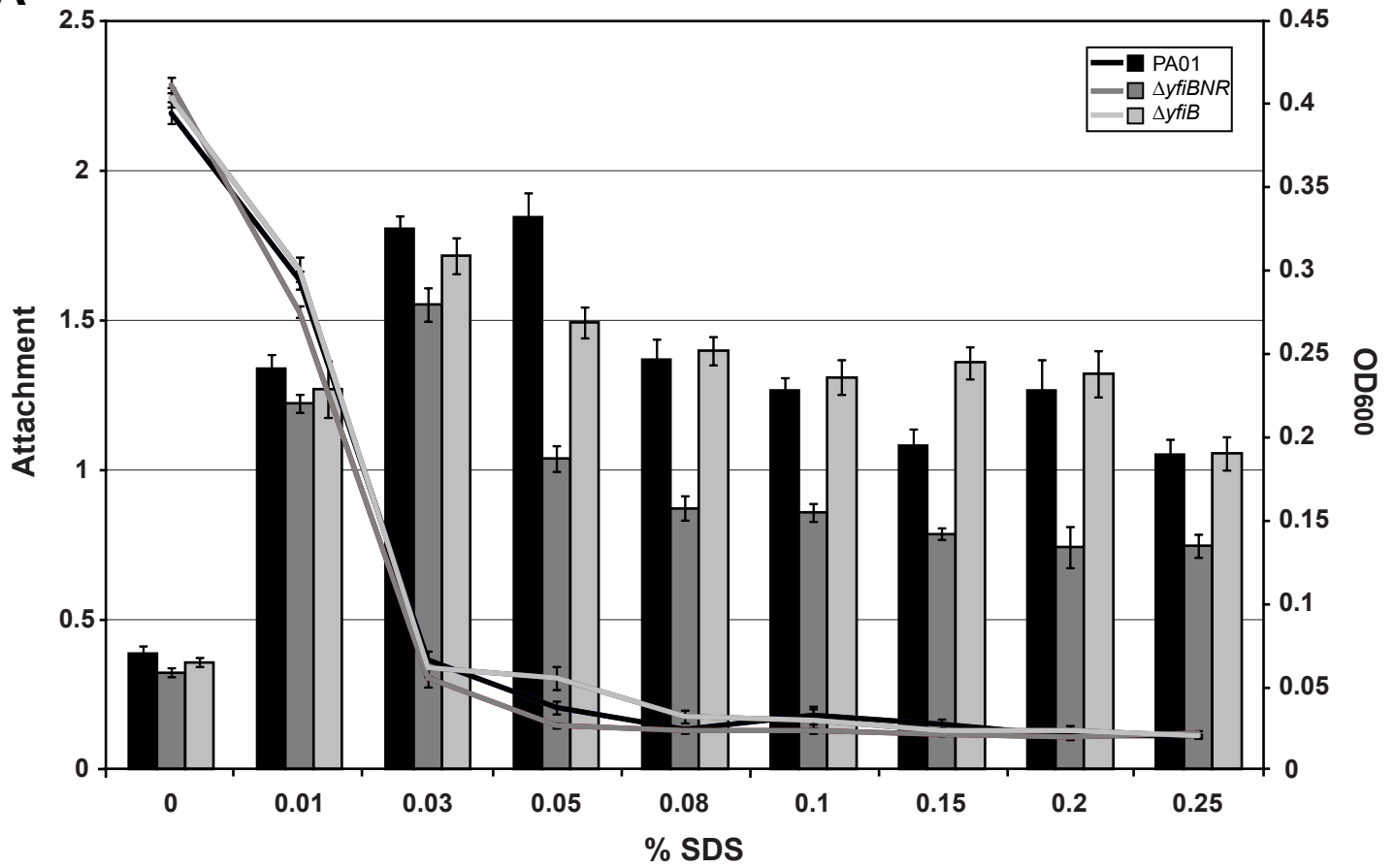

**B**

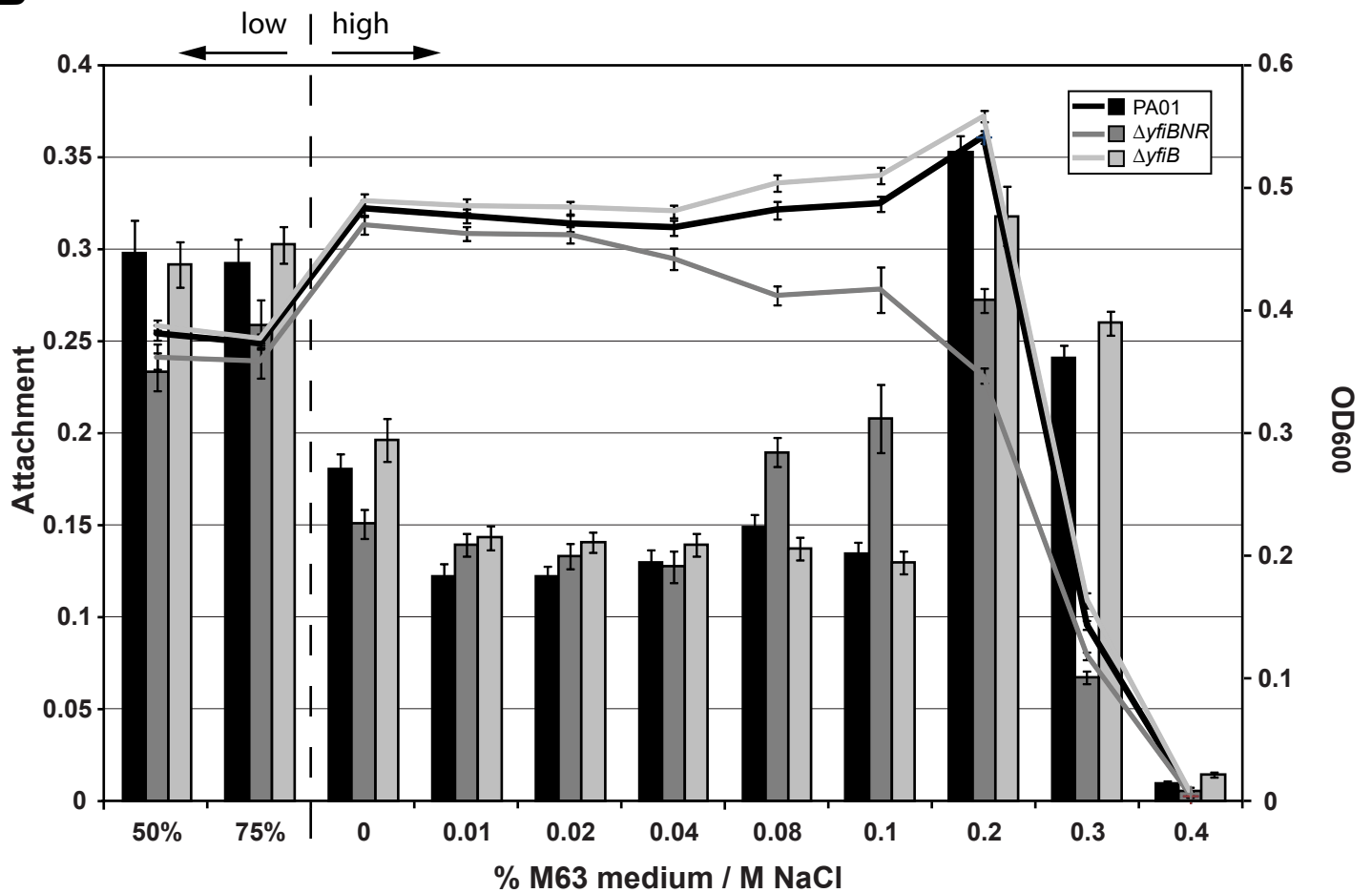

Supplement: Figure S3 — Attachment of Yfi mutants in response to the presence of SDS or changes in osmolarity. A) Attachment on M9 medium with 20 mM Na-succinate and increasing concentrations of SDS is shown relative to PA01. B) Attachment with low and high osmolarity, on M63 medium with 20 mM Na-succinate, 0.2% glucose, and increasing concentrations of NaCl is shown relative to PA01. 50% and 75% indicate dilutions of the medium with distilled water. ΔBNR indicates ΔyfiBNR and ΔB indicates ΔyfiBNR Tn7::yfiNR. Bars represent the level of absolute attachment and curves represent optical density (OD) of total cells with standard errors. (PDF) [file ppat.1002760.s003.pdf]

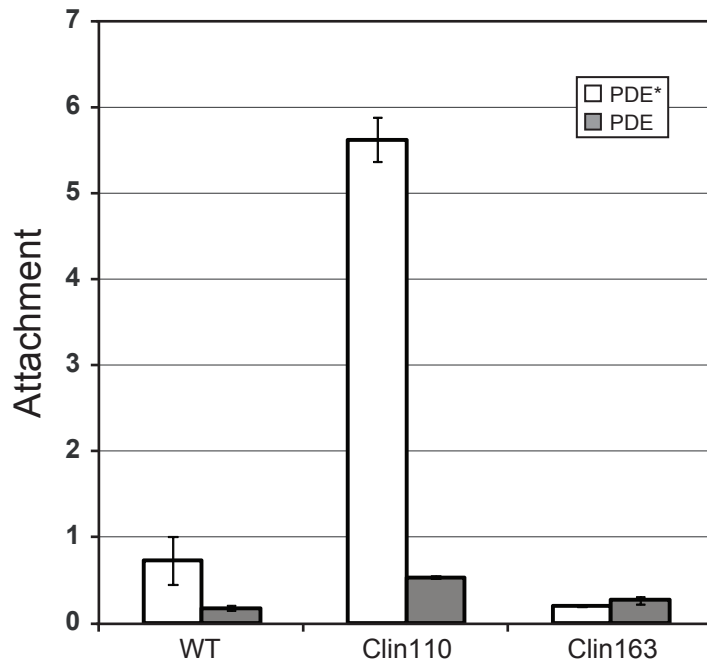

Supplement: Figure S4 — Attachment of clinical iosolates in the presence of a phosphodiesterase. The effect of a PDE, in Clin110 and Clin163, on attachment is shown relative to strains expressing an active site mutant (PDE*). The PDE (PA5295) and the active site mutant are expressed from a vanillate inducible vector (pBV-PA5295 and pBV-PA5295E328A respectively). (PDF) [file ppat.1002760.s004.pdf]
