# Supplementary material for: The YfiBNR Signal Transduction Mechanism Reveals Novel Targets for the Evolution of Persistent Pseudomonas aeruginosa in Cystic Fibrosis Airways
Source: PLoS Pathog. 2012 Jun 14;8(6):e1002760. doi: 10.1371/journal.ppat.1002760 (PMC3375315; doi:10.1371/journal.ppat.1002760)
Supplement: Figure S5 — Sequence conservation across distant YfiN/YfiR homologs. Sequence alignments for YfiR (residues 55–190) and the YfiN PAS domain (residues 61–115), across PA01 and five distant homologs. The five species whose yfiNR genes were compared were: Opitutus terrae PB90-1, Acidobacteria bacterium Ellin345, Geobacter sp. M21, Rhodothermus marinus DSM 4252, and Desulfobacterium autotrophicum HRM2. Fully conserved residues are marked with an asterisk (*), similarities are marked with (:) or (.). Putative hydrophobic binding site residues are enclosed in black boxes. Residues are colored according to the chemical nature of their side chains. (PDF) [file ppat.1002760.s005.pdf]

YfiN PAS domain

```
PA01 YfiN          SYTVEAAVVFGDAQAAEESLALIASSEEVSSAIVYDRQGQTLASWHRESTGPLHL----- 115
opitutis           AINSTAALAFDNPEAAAEILGALRAEPDVAAAVIYTSTGSVFARYP--AKLSAED----- 106
acidobacter        AANSVSALEFNDPESARQTLRALAASPNVEGAGLLTPDGQLFAEYWRSADVRVNV----- 108
rhodothermus       TEHSVAPLVFQDAQAASEVLEGVRSIPAVQIAALYTADGRLFAWYLG--GLPATA----- 108
desulphobacterium  GDNSTAALLFQDDEALENNLHSLAQKTSILTSAIYRADGARVASFSRGGHQQISP----- 111
geobacter          GANSASALVFDDPESAQRTIAPLASKRSILAVYVLTSEGQIMARHVAPEAGNAELPLERL 120
                        :.: * : ::  . :  :     :    :    *  .*
```

YfiR

```
PA01 YfiR          IFSYVRWPKEPAV-----LQLCVVGPTEYADGLLRGMVQANGRRVHAERRAVDNPDLGTL 109
opitutis           FARFVEWPRDVAAQ-ADEVVIGVLGADPFGLYLDEVLRGQKVNGHALVVKRYGSVAEIDR 115
acidobacteria      FGAFVQWPASVAP--SDDFSICVLGRDGFGSVLDSTINGESIEGKKLVALRVNSVREAAQ 117
geobacter          MSKYIEWPAEAFPRTGAPLQICSVGRGPFAAALEQYQ-GKTVLGHQLSLRRLAVGDEPAE 105
rhodothermus       FAQFSTWPEEALGDAATPIRLCILGKDPFGSALEQLQ-GKTVHNRPLEIRHLPVGASARG 102
desulphobacterium  FAKFIKWPGPTFIHEKSPLLIGVMGDDSFADKLGPLT-SRTVRNRPFEIKKLKTSKQAQT 112
                   :  :  **          . :  :*   :.  *       .

PA01 YfiR          CNVIYLGVVDERERQQVFRSLAGHPVLSISERGTECSVGSMFCLNVGGPRITFEANLDSI 169
opitutis           CHVLFMSGSEGERAEPILQALRTRPILTVCDTNAFARHGAMIHLVMDQQRVRLRINLDAA 175
acidobacteria      CRILYISNSEESRLRGILVELAKAPVLTVSDIPHFADRGGMIEFTLQSGRVRFDVNVTRA 177
geobacter          CHVLVVSGVEKRYLAGVLDQARRRDALTVGDIPDFARFGGIIGFVENEGRVRFVINLKAA 165
rhodothermus       CHLVFMGPMPKQVLQTSLKQLQNQPVLTVGEGEDFLKQGGMVRLVEEKNRIQFEINITAA 162
desulphobacterium  CHMVYINTSSSAELSNILEQLKARPIITVGDNNKFAAQGGIIQFIKIRDRLRFIINLDAA 172
                   *.:: :.          :        ::: :       *.:. :     *: :  *:

PA01 YfiR          ARSGVRVHPSVLKLARRQATP---- 190
opitutis           RQAGLVISSKLLRTAEIVTDSEGMR 200
acidobacteria      ERAGLTLSSQLLKVAANVKHDEARD 202
geobacter          QQSRVRISSQLLKLAKLIREGDQ-- 188
rhodothermus       RRAGLQLSARLLRLARIYEEAG--- 184
desulphobacterium  KINQVQIDAQLLSLATELLETKE-- 195
                       : : . :*  *
```
