# Supplementary material for: The YfiBNR Signal Transduction Mechanism Reveals Novel Targets for the Evolution of Persistent Pseudomonas aeruginosa in Cystic Fibrosis Airways
Source: PLoS Pathog. 2012 Jun 14;8(6):e1002760. doi: 10.1371/journal.ppat.1002760 (PMC3375315; doi:10.1371/journal.ppat.1002760)
Supplement: Table S1 — Bacterial strains and plasmids used in this study. (PDF) [file ppat.1002760.s006.pdf]

Table S1. Bacterial strains and plasmids used in this study.

Name	Description	Reference
P. aeruginosa		
PA01	Wild-type *P. aeruginosa*	[1]
Δ*yfiR*	PA01 with *yfiR* deleted	[2]
Δ*yfiNR*	PA01 with *yfiNR* deleted	[2]
Δ*yfiBNR*	PA01 with *yfiBNR* deleted	[2]
Δ*yfiBNR* Tn*7*::*yfiNR*	Δ*yfiBNR* with *yfiNR* genes inserted at the Att::Tn*7* locus	[2]
PA01 *yfiR*-M2	Δ*yfiR* with *yfiR-flag* inserted at the Att::Tn*7* locus	[2]
Δ*yfiBN yfiR*-M2	Δ*yfiBNR* with *yfiR-flag* inserted at the Att::Tn*7* locus	[2]
Δ*yfiN yfiR*-M2	Δ*yfiNR* with *yfiR-flag* inserted at the Att::Tn*7* locus	[2]
PA01 Tn*7*::*yfiRN-flag*	PA01 with *yfiRN-flag* inserted at the Att::Tn*7* locus	This study
Δ*yfiNR* Tn*7*::*yfiRN-flag*	Δ*yfiNR* with *yfiRN-flag* inserted at the Att::Tn*7* locus	This study
Δ*dsbA*	PA01 with *dsbA* deleted	This study
Δ*dsbA* Δ*yfiBNR*	Δ*yfiBNR* with *dsbA* deleted	This study
Δ*dsbA* Δ*yfiB*	Δ*dsbA* Δ*yfiBNR* with *yfiNR* genes inserted at the Att::Tn*7* locus	This study
wspR::Tn*5*	Tn*5* insertion in *wspR*	This study
Δ*dsbA wspR*::Tn*5*	Δ*dsbA* with Tn*5* insertion in *wspR*	This study
Clin110	SCV strain isolated from CF sputum	[2]
Clin163	Smooth strain isolated from CF sputum. Descendent of Clin110	This study
Δ*yfiBNR* Tn*7*::*yfiBNR* Clinical *yfiN* alleles	Δ*yfiBNR* with *yfiBNR* genes inserted at the Att::Tn*7* locus. *yfiN* present in four alleles containing combinations of G173D and Δ255-7 mutations	This study
SCV20265	SCV strain isolated from CF sputum	[3]
WT20265	Smooth precursor of SCV20265, isolated from CF sputum	[3]
E. coli		
DH5α	*endA*1, *hsdR*17(r_K-m_K+), *supE*44, *recA*1, *gyrA* (Nal^r), *relA*1, Δ(*lacIZYA-argF*)U169, *deoR*, Φ80*dlac*Δ*(lacZ)M15*	[4]
DY330	W3110 *ΔlacU169 gal490 λcI857 Δ(cro-bioA)*	[5]
XL1-red	F- endA1 *gyrA*96(*nalR*) *thi*-1 *relA*1 *lac glnV*44 *hsdR*17(rK- mK+) *mutS mutT mutD*5 Tn10	Stratagene
**Plasmids**		
pME6032	Tet^R, P_K, 9.8 kb pVS1 derived shuttle vector	[6]
pME6032-Gm	Gm^R, pME6032 based vector, Tet cassette replaced with *aacCI* gentamycin resistance cassette	This study
pME-*araC*	Tet^R, pME6032 with the lac-promoter and *lacI* replaced with the *araC*-pBAD promoter fragment from pBAD18s	[2]
pME-*araC-yfiR*	Tet^R, pME-*araC* containing YfiR under control of ara-promoter as EcoRI-BglII fragment	[2]
pME6032-*yfiB*	pME6032 with *yfiB* as *EcoRI-BglI* fragment	[2]
pBV-PA5295	pBV-MCS4 with PA5295 under the control of vanillate promoter	[2]

pBV-PA5295$_{E328A}$	pBV-MCS4 with PA5292$_{E328A}$ under the control of vanillate promoter	[2]
Activated *yfiB* mutants	20 pME6032-*yfiB* variants with activating mutations in *yfiB* (see Table 3)	This study
pGm-*yfiprom-N*	pME6032-Gm with *yfiN* as *Bam*HI-blunt fragment, *yfiN* expressed from the native promoter	This study
pGm-*yfiprom-N -constructs*	pME6032-Gm with yfi*N* alleles as *Bam*HI-*Nco*I fragments, containing single point mutations	This study
YfiR-insensitive YfiN mutants	20 pGm-*yfiprom-N* variants with YfiR-insensitive mutations in *yfiN* (see Table 1 and Fig.7C)	This study
pMR-*yfiR-flag*	pMR20-*yfiR-M2* with lac promoter replaced by FRT scar sequence	This study
Compensatory YfiR mutants	14 pMR-*yfiR-flag* variants with compensatory mutations in *yfiR* (see Table 2 and Fig. 7C)	This study
pME-*yfiB-PG* - constructs	pME6032-*yfiB* with *yfiB* D102A,G105A (± F48S, L43P) as *Eco*RI-*Asc*I fragments	This study
pME-*yfiB-LA* - constructs	pME6032 containing *yfiR* residues 1-42 fused to *yfiB* (± D102A,G105A) residues 26-169 as *EcoRI-BglI* fragment	This study
pME-*yfiB* short/long constructs	pME6032 with *yfiB* short/long linker alleles as *EcoRI-BglI* fragments	This study
pME-*yfiB* constructs	pME6032 with *yfiB* alleles as *Eco*RI-*Bgl*II fragments, containing single point mutations	This study
pME-*dsbA*	pME6032 with *dsbA* as *Eco*RI-*Kpn*I fragment	This study
pME3087	Tet^R, suicide vector for allelic replacement; *ColE1*-replicon, *IncP-1*, *Mob*	[7]
pME3087-Δ*dsbA*	pME3087 with *dsbA* deletion cassette as *Hind*III-*Bam*HI fragment	This study
pMR20	Tet^R, low copy number and broad host-range vector	[8]
pMR20-*yfiBNR*	pMR20 with *yfiBNR* as *Hind*III-*Bam*HI fragment	[2]
pMR20-*yfiR-M2*	pMR20 with *yfiR-flag* as *Hind*III-*Bam*HI fragment	[2]
pMR20-*yfiR-M2 -constructs*	pMR20 with *yfiR-flag* alleles as *Hind*III-*Bam*HI fragments, containing single point mutations	This study
pMR20-*yfiRN-flag*	pMR20 with *yfiRN-flag* as *Hind*III-*Bam*HI fragment	This study
pUC18T-mini-Tn*7*T-Gm	Amp^R, Gm^R, Tn*7* insertion vector	[9]
pTn*7*-*yfiR-M2*	pUC18T-mini-Tn*7*T with *yfiR-flag* as *Hind*III-*Bam*HI fragment	[2]
pTn*7*-*yfiRN-flag*	pUC18T-mini-Tn*7*T with *yfiRN-flag* as *Hind*III-*Bam*HI fragment	This study
pTn*7*-*yfiBNR*	Vector for complementation of entire *yfiBNR* operon at Tn*7* locus	[2]
pTn*7*-*yfiBN-G173D-R*	Vector for complementation of *yfiBNR* operon with *yfiN* G173D mutation	This study
pTn*7*-*yfiBN-Δ255-7-R*	Vector for complementation of *yfiBNR* operon with *yfiN* Δ255-7 mutation	This study
pTn*7*-*yfiBN20265-R*	Vector for complementation of *yfiBNR* operon with SCV20265 *yfiN* allele	This study
pTNS2	Amp^R, helper plasmid for Tn*7* integration events	[9]
pFLP2	Amp^R, FRT cassette excision vector	[10]

References

1. Holloway BW (1955) Genetic recombination in Pseudomonas aeruginosa. J Gen Microbiol 13: 572-581.
2. Malone JG, Jaeger T, Spangler C, Ritz D, Spang A, et al. (2010) YfiBNR mediates cyclic di-GMP dependent small colony variant formation and persistence in Pseudomonas aeruginosa. PLoS Pathog 6: e1000804.
3. Meissner A, Wild V, Simm R, Rohde M, Erck C, et al. (2007) Pseudomonas aeruginosa cupA-encoded fimbriae expression is regulated by a GGDEF and EAL domain-dependent modulation of the intracellular level of cyclic diguanylate. Environ Microbiol 9: 2475-2485.
4. Woodcock DM, Crowther PJ, Doherty J, Jefferson S, DeCruz E, et al. (1989) Quantitative evaluation of Escherichia coli host strains for tolerance to cytosine methylation in plasmid and phage recombinants. Nucleic Acids Res 17: 3469-3478.
5. Yu D, Ellis HM, Lee EC, Jenkins NA, Copeland NG, et al. (2000) An efficient recombination system for chromosome engineering in Escherichia coli. Proc Natl Acad Sci U S A 97: 5978-5983.
6. Heeb S, Itoh Y, Nishijyo T, Schnider U, Keel C, et al. (2000) Small, stable shuttle vectors based on the minimal pVS1 replicon for use in gram-negative, plant-associated bacteria. Mol Plant Microbe Interact 13: 232-237.
7. Voisard C, Bull CT, Keel C, Laville J, Maurhofer M, et al. (1994) Biocontrol of root diseases by Pseudomonas fluorescens CHA0: current concepts and experimental approaches. In: O'Gara F, Dowling DN, Boesten B, editors. Molecular Ecology of Rhizosphere Microorganisms. Weinheim, Germany: Wiley-VCH. pp. 67-89.
8. Roberts RC, Mohr CD, Shapiro L (1996) Developmental programs in bacteria. Curr Top Dev Biol 34: 207-257.
9. Choi KH, Gaynor JB, White KG, Lopez C, Bosio CM, et al. (2005) A Tn7-based broad-range bacterial cloning and expression system. Nat Methods 2: 443-448.
10. Hoang TT, Karkhoff-Schweizer RR, Kutchma AJ, Schweizer HP (1998) A broad-host-range Flp-FRT recombination system for site-specific excision of chromosomally-located DNA sequences: application for isolation of unmarked Pseudomonas aeruginosa mutants. Gene 212: 77-86.
