# Supplementary material for: The YfiBNR Signal Transduction Mechanism Reveals Novel Targets for the Evolution of Persistent Pseudomonas aeruginosa in Cystic Fibrosis Airways
Source: PLoS Pathog. 2012 Jun 14;8(6):e1002760. doi: 10.1371/journal.ppat.1002760 (PMC3375315; doi:10.1371/journal.ppat.1002760)
Supplement: Table S2 — Primers used in this study. (PDF) [file ppat.1002760.s007.pdf]

Table S2. Primers used in this study.

Primer Name	Role	Sequence
A	*yfiN*-FLAG For	GAAAAGGAGATAGACAGTGCTACCCCAGAGGCTCCACCCAAGGACTACAAAGACCATGACGG
B	*yfiN*-FLAG Rev	TTACTCGGCGGGAACGATGATGGCGACGCGGCGGTTCTCGCATATGAATATCCTCCTTAG
C	Chlor For	CGCGCGTTGGCCGATTCATTAATGCAGCTGGCACGACAGGTGTGTAGGCTGGAGCTGCTTC
D	Chlor Rev	TCTATTAAAGGTCATTCAAAAGGTCATCCACCGGATCTATCATATGAATATCCTCCTTAG
E	Gent For	GAGGATCCATTAAGCATTCTGCCGAC
F	Gent Rev	CGTCTAGATGACGGAACAGCGGGAAG
G	*yfiR* For	CCGAAGCTTGCCGCCCCCTG
H	*yfiN* Rev	GCGGATCCTCTACTTGGGTGG
I	*yfiB* For	GAGAATTCGTGCTACCCCAG
J	*yfiB* Rev	GAGGATCCGATTACTCGGCG
K	DsbA For	GATCAGAATTCTAGGAGTGAACGATGC
L	DsbA Rev	GATCGGTACCTTGCCTACTTCTTGG
M	*yfiN* Seq 1	CCCAACCAGCAACTGATC
N	*yfiN* Seq 2	TGCTTTCCCGATGCCAAC
O	*yfiN* Seq 3	GTCTATCTCCTTTTCTTC
P	*yfiR* Rev	GAGGATCCATCACGGAGTGGC
Q	Δ*dsbA* UpFor	GATCGGAACTAAGCTTGATGTG
R	Δ*dsbA* UpRev	GGAAGAAATGGCCCATATGCTCGCCG
S	Δ*dsbA* DownFor	GGTGAGAATCATATGACGCATCGTTCACTCCTAGG
T	Δ*dsbA* DownRev	GTGAGTCTGCTGTGGATCCTGGGTCTG
U	*yfiB* PG- Rev	TCGGCGCGCCGTTCGGAGAGCTTTTGATTGTAGCCTTCGTCGGCGTAGTTGGCGGTGTGGCCTTC
V	*yfiR* LAfus For	CTCTCTCAATTGTTGCCTTCGTTGCCCAC
W	*yfiB* LAfus For	CTCGACGTCGTGCCAGACCAAGC
X	*yfiB* long For	GCCTGAGCACCAAGCCGCCGCAGACCGGCCTGAGCGCCGAGCAGATCG
Y	*yfiB* long Rev	GTCTGCGGCGGCTTGGTGCTCAGGCCGGTCTGCGGCGGCTTGGTCTGG
Z	*yfiB* short For	CTGGCCGGTTGCCAGACCGGCCTGAGCGCCGAGCAGATCG
AA	*yfiB* short Rev	CGATCTGCTCGGCGCTCAGGCCGGTCTGGCAACCGGCCAG
AB	*yfiB G70A* For	CTTGTGCTGGGCCTAGCCGGTTGCCAG
AC	*yfiB G70A* Rev	CTGGCAACCGGCTAGGCCCAGCACAAG
AD	*yfiB A224C* For	GTCTCAATCCCGCCAGCCGCAACAC
AE	*yfiB A224C* Rev	GTGTTGCGGCTGGCGGGATTGAGAC
AF	*yfiB G254A* For	CCAAGATCGCCCACGCCCTGCTC
AG	*yfiB G254A* Rev	CGAGCAGGGCGTGGGCGATCTTGG
AH	*yfiB* For	GAAACAGAATTCGTGCTACCCCAGAG
AI	*yfiB* Rev	GATCAGATCTGATTACTCGGCGGGAACGATGATGG
AJ	*yfiN A301C* For	GACAGCCCCTGGCCAGTTGGCATC
AK	*yfiN A301C* Rev	CAACTGGCCAGGGGCTGTC
AL	*yfiN G667A* For	CTGGGCGAGAACTTCAACG
AM	*yfiN G667A* Rev	CGTTGAAGTTCTCGCCCAG
AN	*yfiN C679A* For	GGACTTCAACGCCATGCTCGACG
AO	*yfiN C679A* Rev	CGTCGAGCATGGCGTTGAAGTCC
AP	*yfiN A824G* For	CTGCGCGATGCCAGCGAGCATCG
AQ	*yfiN A824G* Rev	CTCGCGATGCTCGCTGGCATCGCGCAG
AR	*yfiN* For	GATCCCATGGTTGCCGCCCCCTGATAAAGG
AS	*YfiN* Rev	CTTAATGGATCCTCTACTTGGGTGGAGC
AT	*yfiR T76C* For	CTGGCGGTTTCCCGCCTGGG
AU	*yfiR T76C* Rev	CCCAGGCGGGAAACCGCCA
AV	*yfiR G383A* For	CAGCAGGTGTTCCACAGCCTGG
AW	*yfiR G383A* Rev	CCAGGCTGTGGAACACCTGC
AX	*yfiR G391A* For	GTGTTCCGCAGCCTGACCGGGCATC
AY	*yfiR G391A* Rev	GATGCCCGGTCAGGCTGCGGAACA
AZ	*yfiR-mut* For	GATCAAGCTTGCCGCCCCCTGATAAAGGTC
BA	*yfiR*-FLAG Rev	CCATGAGCTCGGATCCGCGTTACTATTTATCGTCGTCATC
