# Supplementary material for: The YfiBNR Signal Transduction Mechanism Reveals Novel Targets for the Evolution of Persistent Pseudomonas aeruginosa in Cystic Fibrosis Airways
Source: PLoS Pathog. 2012 Jun 14;8(6):e1002760. doi: 10.1371/journal.ppat.1002760 (PMC3375315; doi:10.1371/journal.ppat.1002760)
Supplement: Table S3 — Single nucleotide polymorphism (SNP) positions in the sequences of clinical SCVs, compared to P. aeruginosa PA01. (PDF) [file ppat.1002760.s008.pdf]

Table S3. Single nucleotide polymorphism (SNP) positions in the sequences of clinical SCVs, compared to *P. aeruginosa* PA01.

Locus	Nucleotide position	AA position	PA01 nucleotide	SNP	PA01 AA	SNP AA	SCV Pool 1	SCV Pool 2	SCV Pool 3	WT20265	SCV20265	Clin110 (SCV)	Clin163 (Smooth)	Notes
Upstream	-182	0	G	A			X	X						
Upstream	-171	0	G	A				X						
Upstream	-138	0	G	T			X	X	X					
Upstream	-115	0	G	A			X	X	X					
Upstream	-109	0	T	C			X	X	X					
Upstream	-34	0	C	T			X	X	X					
**YfiR**	76	26	T	C	C	R		X						SCV inducing
**YfiR**	121	41	A	G	I	V	X	X	X	X	X	X	X	Untested
**YfiR**	138	46	T	C	N	N	X	X	X					
**YfiR**	192	64	G	A	E	E	X	X	X					
**YfiR**	201	67	C	T	V	V		X	X					
**YfiR**	222	74	A	C	G	G		X						
**YfiR**	383	128	G	A	R	H	X	X						No effect seen
**YfiR**	391	131	G	A	A	T	X	X						No effect seen
**YfiR**	405	135	G	C	V	V	X	X	X	X	X	X	X	
**YfiR**	423	141	C	T	R	R	X	X	X	X	X	X	X	
**YfiR**	471	157	C	T	G	G	X	X	X					
**YfiN**	63	21	G	A	A	A	X		X	X	X	X	X	
**YfiN**	108	36	G	A	A	A		X						
**YfiN**	144	48	T	C	Y	Y	X	X	X					
**YfiN**	147	49	C	G	A	A	X	X						
**YfiN**	195	65	G	A	E	E	X	X		X	X	X	X	
**YfiN**	201	67	G	A	A	A	X			X	X	X	X	
**YfiN**	234	78	G	A	E	E	X	X						
**YfiN**	259	87	G	A	E	K						X	X	SCV inducing
**YfiN**	294	98	G	A	Q	Q		X						
**YfiN**	297	99	A	G	G	G		X						
**YfiN**	301	101	A	C	T	P		X						No effect seen
**YfiN**	312	104	T	C	S	S		X						
**YfiN**	438	146	G	A	K	K		X	X					
**YfiN**	474	158	C	A	T	T	X		X	X	X	X	X	
**YfiN**	518	173	G	A	G	D	X				X			SCV inducing
**YfiN**	534	178	G	A	S	S		X						
**YfiN**	667	223	G	A	D	N	X							SCV inducing
**YfiN**	679	227	C	A	L	M	X							SCV inducing
**YfiN**	712	238	C	T	L	L		X						
**YfiN**	765	255-7	TSL deleted								X			Inactivating

**YfiN**	824	275	A	G	N	S		X	X					No effect seen
**YfiN**	831	277	T	C	H	H		X	X					
**YfiN**	849	283	G	A	V	V		X	X					
**YfiN**	870	290	C	T	R	R	X	X						
**YfiN**	984	328	C	T	G	G	X	X	X					
**YfiN**	985	329	G	T	G	C							X	Inactivating
**YfiN**	1002	334	C	G	V	V	X	X	X					
**YfiN**	1011	337	A	G	A	A			X					
**YfiN**	1101	367	T	G	T	T	X	X	X	X	X	X	X	
**YfiN**	1113	371	A	G	S	S	X	X	X	X	X	X	X	
**YfiN**	1146	382	T	C	H	H	X	X	X					
**YfiN**	1188	396	G	A	A	A						X	X	
**YfiN**	1222	408	C	A	R	R		X						
**YfiB**	36	12	C	T	L	L	X	X	X	X	X	X	X	
**YfiB**	66	22	C	T	G	G	X	X	X					
**YfiB**	69	23	G	C	L	L			X					
**YfiB**	70	24	G	A	A	T	X	X	X					No effect seen
**YfiB**	87	29	G	A	K	K		X	X					
**YfiB**	180	60	G	C	S	S	X	X	X	X				
**YfiB**	207	69	C	A	L	L	X	X	X	X	X	X	X	
**YfiB**	224	75	A	C	D	A	X		X					No effect seen
**YfiB**	243	81	C	G	T	T	X	X	X					
**YfiB**	254	85	G	A	R	H		X	X					No effect seen
**YfiB**	259	87	C	T	L	L	X	X	X					
**YfiB**	330	110	T	C	N	N		X	X					
**YfiB**	333	111	A	G	Q	Q		X	X					
**YfiB**	363	121	G	A	V	V		X						
**YfiB**	372	124	A	G	V	V		X						
**YfiB**	420	140	T	G	L	L		X						
**YfiB**	435	145	C	G	P	P	X	X		X	X	X	X	

Non-synonymous mutations are marked in blue. ‘X’ indicates the presence of a particular mutation in the sequence indicated. The YfiR I41V substitution was ubiquitous among the sequenced strains, suggesting that valine is the ancestral residue at this position and a V to I substitution occurred in PA01.
