# Supplementary material for: The YfiBNR Signal Transduction Mechanism Reveals Novel Targets for the Evolution of Persistent Pseudomonas aeruginosa in Cystic Fibrosis Airways
Source: PLoS Pathog. 2012 Jun 14;8(6):e1002760. doi: 10.1371/journal.ppat.1002760 (PMC3375315; doi:10.1371/journal.ppat.1002760)
Supplement: Table S4 — Identification of YfiBNR homologs in other systems. (PDF) [file ppat.1002760.s009.pdf]

Table S4. Identification of YfiBNR homologs in other systems.

Genus	*yfiBNR*	*yfiRN, yfiB* elsewhere	*yfiNB,* no *yfiR*	Notes
Accumulibacter	0	1	0	
Acidobacteria	0	1	0	'YfiN' has histidine kinase output domain
Acinetobacter	7	0	0	
Aeromonas	2	0	0	
Alcanivorax	0	0	1	
Alicycliphilus	1	0	0	
Alkalilimnicola	0	0	1	
Burkholderia	9	0	0	
Citrobacter	2	0	0	
Cupriavidus	2	0	0	
Dechloromonas	0	1	0	'YfiN' has histidine kinase output domain, operon contains cdG genes
Delftia	1	0	0	
Desulfobacterium	0	1	0	'YfiN' output is a BarA homolog
Desulfovibrio	0	0	1	
Dickeya	0	2	0	No YfiB present in operon, otherwise similar to PA01 YfiNR
Edwardsiella	1	0	0	
Enterobacter	3	0	0	
Escherichia	32	0	0	
Geobacter	0	1	1	'YfiN' has GGDEF-EAL output domains
Hahella	0	0	1	
Kangiella	0	0	1	
Klebsiella	4	0	0	
Leptothrix	0	1	0	
Methylotenera	0	0	1	
Methylovorus	2	0	0	
Opitutus	0	1	0	'YfiN' has histidine kinase output domain
Pantoea	2	0	0	
Polaromonas	0	1	0	'YfiN' has MCP output domain
Pseudomonas	12	0	0	
Ralstonia	2	1	0	
Rhodothermus	0	1	0	'YfiN' has histidine kinase output domain
Salmonella	0	14	0	No YfiB present in operon, otherwise similar to PA01 YfiNR
Serratia	1	0	0	
Shigella	1	5	0	Possibly due to system degradation in some cases
Sideroxydans	0	1	0	'YfiN' has GGDEF-EAL output domains
Sphingobium	0	1	0	
Sphingopyxis	1	0	0	
Sulfuricurvum	0	0	1	
Teredinibacter	0	1	0	
Thauera	0	1	0	'YfiN' has HPT output domain, in an operon with 2 YfiR homologs
Thioalkalivibrio	0	0	1	
Xanthomonas	0	0	1	
Xenorhabdus	1	0	0	
Yersinia	13	0	0	
**Total**	***99***	***35***	***10***	
